# Dispersion tuning and route reconfiguration of acoustic waves in valley topological phononic crystals

**DOI:** 10.1038/s41467-020-14553-0

**Published:** 2020-02-07

**Authors:** Zhenhua Tian, Chen Shen, Junfei Li, Eric Reit, Hunter Bachman, Joshua E. S. Socolar, Steven A. Cummer, Tony Jun Huang

**Affiliations:** 1 Department of Mechanical Engineering and Materials Science, Duke University, Durham, NC 27708 USA; 2 Department of Electrical and Computer Engineering, Duke University, Durham, NC 27708 USA; 3 Department of Physics, Duke University, Durham, NC 27708 USA; 4 Present Address: Department of Aerospace Engineering, Mississippi State University, Starkville, MS 39762 USA

**Keywords:** Photonic crystals, Acoustics, Topological insulators

## Abstract

The valley degree of freedom in crystals offers great potential for manipulating classical waves, however, few studies have investigated valley states with complex wavenumbers, valley states in graded systems, or dispersion tuning for valley states. Here, we present tunable valley phononic crystals (PCs) composed of hybrid channel-cavity cells with three tunable parameters. Our PCs support valley states and Dirac cones with complex wavenumbers. They can be configured to form chirped valley PCs in which edge modes are slowed to zero group velocity states, where the energy at different frequencies accumulates at different designated locations. They enable multiple functionalities, including tuning of dispersion relations for valley states, robust routing of surface acoustic waves, and spatial modulation of group velocities. This work may spark future investigations of topological states with complex wavenumbers in other classical systems, further study of topological states in graded materials, and the development of acoustic devices.

## Abstract (web summary)

The valley degree of freedom gives additional flexibility to tunable phononic and photonic crystals. Here, the authors realise a honeycomb phononic structure where both the size of the cavities and of the air channel can be actively tuned, allowing several functionalities in a broad frequency range.

## Introduction

Topological materials that exhibit a valley degree of freedom (DOF) enable a way to transport information and energy^1–11^ and are attracting a growing interest in condensed matter physics^6^. Recently, the concept of valley topological phases has been extended to classical bosonic systems, inspiring various ways of manipulating classical waves using periodic structures, such as electromagnetic waves with photonic crystals^12–18^ or acoustic/elastic waves with phononic crystals (PCs)^19–33^. The valley Hall phases for classical waves are generally realized by creating a periodic lattice that breaks a mirror^19–23^ or inversion^25–31^ symmetry of a two-dimensional (2D) honeycomb lattice. In such structures, the eigenmodes at inequivalent valleys naturally possess opposite polarizations^24,27^, giving rise to topological transport that does not require strong spin-orbit interactions. At interfaces between distinct valley Hall phases, edge states emerge^20,26^ that are immune to backscattering at defects^22,23^, imperfections^28,34^, and sharp corners^19,30^ in the wave path^20,33^.

Despite intensive research on tunable acoustic systems^34–47^, including metamaterials^35–39^, metasurfaces^40–42^, recently reported topological insulators^43^, and rotatable-unit-based valley PCs^34^, tuning the dispersion (frequency, wavenumber, and slope) of topological states for acoustic waves is challenging. For previously reported topological acoustic systems, including two of the most recent designs^34,43^, it remains difficult to continuously tune the frequencies of topological states over a wide range, because the frequencies are inherently tied to fixed lattice constants and unit cell designs. The frequency tunability could be useful for wideband tunable acoustic devices. Moreover, previous topological acoustic systems cannot continuously tune the dispersion slope, which would be useful for controlling the group velocities of acoustic topological states, and they afford limited control over the imaginary part of the wavenumber, which is associated with an exponential decay of waves that is not manifested in many topological acoustic systems. Although systems with topological bands in the real wavenumber—frequency domain have been intensively studied and developed for applications^19–33^, there have been few studies on acoustic systems with topological bands in the complex wavenumber—frequency domain. Similarly, few studies have explored acoustic topological states in PCs with chirped or other graded structures.

Topological acoustic systems have been designed with various functionalities, such as robust sound transport^48–50^, directional antennas^22^, negative refraction^51^, and acoustic delay lines^34^. Nevertheless, some potentially important controllable functionalities have not been demonstrated. Examples include tuning the dispersion of surface acoustic waves (SAWs), tailoring the group velocity of an edge mode in the space-frequency domain, slowing the group velocity of an edge mode to zero, and rainbow trapping of an edge mode with the energy of different frequency components accumulated at different locations. The development of a phononic topological system with multiple tunable parameters that enable the exploration of many different functionalities in one platform could save time and reduce costs associated with fabricating and testing many individual, non-configurable designs.

Here, we present tunable valley PCs composed of hybrid channel-cavity cells with three tunable parameters (channel height and depths of two cavities) for dispersion tuning and route reconfiguration of acoustic waves. The tunable PCs allow for investigating topological physics in PCs in different dynamical regimes and realizing multiple functionalities using a single, reconfigurable platform. We demonstrate experimentally with simulations the tuning of multiple features of dispersion relations, including Dirac point frequencies, band-edge frequencies, imaginary parts of wavenumbers, edge mode frequencies, edge mode group velocities, and edge mode attenuation. Experimental and numerical studies reveal that our PCs support acoustic topological states with complex wavenumbers, and we describe the topological bands, Dirac degeneracies, and edge modes in the complex wavenumber—frequency domain. To further establish the flexibility of our platform, we experimentally demonstrate frequency tuning and robust routing of the valley Hall edge mode of spoof SAWs. Finally, by realizing chirped valley PCs, we experimentally illustrate the modulation of the edge mode group velocity in the space-frequency domain, slowing the group velocity to zero, and rainbow guiding of edge waves. This study represents an advance in engineering tunable PCs with an array of potentially useful properties. The scope of possibilities also suggests avenues for further scientific studies of topological systems with complex wavenumbers and systems with graded structures.

## Results

### Valley topological PCs with hybrid channel-cavity cells

**Fig. 1:**
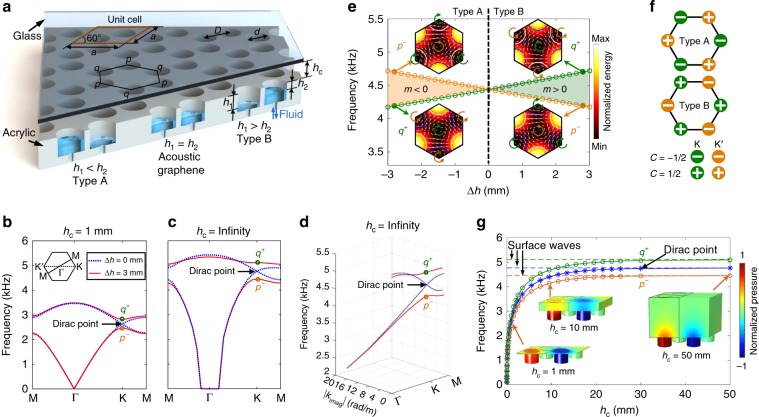
Schematic of valley topological phononic crystals and their dispersion curves. **a** Schematic of the phononic crystals (PCs) composed of a glass ceiling and an acrylic plate with cylindrical cavities distributed in honeycomb lattices. The unit cell (marked by a parallelogram) contains two cavities with the same diameter *d*. The effective depths *h*_1_ and *h*_2_ of cavities can be changed by pumping water into the cavities. When *h*_1_ = *h*_2_, the PCs are considered as an acoustic analogue of graphene. When *h*_1_ < *h*_2_ and *h*_1_ > *h*_2_, t*h*e configurations are denoted as type A and B PCs, respectively. **b** Dispersion curves for valley PCs, when the channel height *h*_c_ is 1 mm. Inset: first Brillouin zone (FBZ). Figure **b** shows a Dirac degeneracy at the K point, when *h*_1_ = *h*_2_ = 11.5 mm. A complete band gap is opened, when *∆h* = *h*_1_ − *h*_2_ = 3 mm. **c** Dispersion curves for valley PCs, when *h*_c_ = infinity. **d** Three-dimensional (3D) view of dispersion curves in the complex wavenumber – frequency domain. Figure **c** can be considered as a projection of the 3D view in the real wavenumber part—frequency domain. **e** Phase diagram revealed by the order of band-edge frequencies locked with specific vortex features (insets), when *h*_c_ = 10.5 mm. The color and white arrows in insets indicate acoustic energy and intensities, respectively. The blue and white arcs represent cavities with depths of 10 and 13 mm. Different acoustic insulating phases are characterized by the signs of the effective mass *m*. **f** Theoretical valley Chern numbers for type A and B PCs. **g** Frequency variations of the Dirac, *p*^−^, and *q*^+^ points with respect to the channel height *h*_c_, when the difference *∆h* is 3 mm. Insets: pressure fields for the *p*^−^ mode. The *q*^+^ mode’s pressure fields are given in Supplementary Fig. 3. The frequencies gradually increase and approach frequencies for surface acoustic waves (SAWs), as *h*_c_ increases. The evolutions of pressure fields and waves frequencies reveal the transition from waveguide acoustic waves to SAWs as the channel height increases.

Figure 1a shows a schematic diagram of the designed valley topological PCs, which are composed of a glass ceiling and an acrylic plate with cylindrical cavities distributed in graphene-like 2D honeycomb lattices^52^. The acoustic waves are confined for 2D propagation in the space between the glass ceiling and the acrylic plate. Each unit cell contains two cylindrical cavities with the same diameter *d* of 8.7 mm, which are *D* = 21.1 mm away from each other. The depths *h*_1_ and *h*_2_ of two cavities can be controlled by pumping water into/out of the cavities through ports connected to the their bottoms. When *h*_1_ = *h*_2_ = 11.5 mm, the dispersion for waves in the PCs features Dirac degeneracies at the K and K′ points (blue dotted lines in Fig. 1b–d). By breaking the inversion symmetry (i.e., creating the type A PCs with *h*_1_ < *h*_2_ or type B PCs with *h*_1_ > *h*_2_ in Fig. 1a), the Dirac degeneracies are removed and a complete bandgap is opened (red solid lines in Fig. 1b–d). The insets in Fig. 1e present simulated acoustic energy and intensity fields for the two states labelled by *p*^*−*^ and *q*^+^ points on the first two bands when *h*_1_ ≠ *h*_2_. The acoustic intensity fields for *p*^*−*^ and *q*^+^ modes show typical vortex profiles with anticlockwise and clockwise energy flows (pseudospins) around the field centers, respectively. The anticlockwise and clockwise pseudospins can be further confirmed by the temporal evolution of pressure fields in a period from *t* = 0 to 2π*/ω* (Supplementary Fig. 1). In addition, the intensity fields for the *p*^*−*^ mode in Fig. 1e indicate clockwise energy flows around three vertices labeled with “*p*” in a hexagon unit (Fig. 1a). The intensity fields for the *q*^+^ mode in Fig. 1e show anticlockwise energy flows around three vertices labeled with “*q*” in a hexagon unit (Fig. 1a).

To reveal the acoustic valley Hall phase transition, the evolution of band-edge frequencies *ω*^p*−*^ and *ω*^q*+*^ at *p*^*−*^ and *q*^+^ modes versus the difference *∆h* = *h*_1_ − *h*_2_ is plotted in Fig. 1e. The evolution clearly shows that the bandgap width increases continuously as *∆h* varies from zero. Figure 1f shows the valley Chern numbers that characterize the distinct valley-dependent behaviors (Methods). For type A PCs with *∆h* < 0, the theoretical valley Chern numbers at the K and K′ points are −1/2 and 1/2, respectively; for type B with *∆h* > 0, these are reversed. These features offer the potential to achieve valley Hall edge states at the interface of type A and B PCs. Moreover, by controlling the difference in *∆h*, the band gap between *p*^*−*^ and *q*^+^ modes can be tuned. To evaluate the change of intervalley mixing induced by the increase of |*∆h*|, valley Chern numbers are calculated by integrating the Berry curvature over a finite square around the valley points with *δk*_*x*_ and *δk*_*y*_ in the range $\left( {{\textstyle{{ - {\uppi}} \over {2a}}},{\textstyle{{\uppi }} \over {2a}}} \right)$^29^. As the inversion symmetry breaking becomes stronger (increase of |*∆h*|), the |*C*_K(K′)_| calculated through integration decreases (Supplementary Fig. 1c). This means that the Berry curvature becomes less localized at K (K′) points and the intervalley mixing becomes stronger^29^.

Another tunable parameter in our acoustic device is the channel height *h*_c_. When the channel height is small (for example *h*_c_ = 1 mm), acoustic waves can strongly interact with the ceiling. For this case, the acoustic waves can be considered as waveguide acoustic waves (WAWs), as the wave energy is confined and guided by the space (waveguide) between the top ceiling and the bottom cavity array (Fig. 1g, inset for *h*_c_ = 1 mm). The topological bands and Dirac cones for WAWs are in the real wavenumber—frequency domain, as shown by the dispersion curves for *h*_c_ = 1 mm (Fig. 1b). When the channel height is very large (for example *h*_c_ ≥ 50 mm), the effect of the top ceiling on acoustic waves is negligible. Hence, the generated acoustic waves can be considered as SAWs that propagate along the bottom cavity array (Fig. 1g, inset for *h*_c_ = 50 mm).

We find that the topological bands for SAWs are in fact in the complex wavenumbers (*k* + *i∙k*_imag_)—frequency domain, as shown in a 3D view of dispersion curves for *h*_c_ = infinity (Fig. 1d). A projection of the 3D view in the real wavenumber part-frequency domain is given in Fig. 1c; a projection in the complex wavenumber space is given in Supplementary Fig. 2a. The 3D view and projections show that band crossing at the Dirac point occurs in the complex wavenumber-frequency domain, when *∆h* = 0 mm (blue dotted lines). Since the imaginary part (*k*_imag_) of the wavenumber is associated with wave attenuation, the intersection in the complex wavenumber domain means that the two SAW modes have the same attenuation at the Dirac point (Supplementary Fig. 2b). This attenuation is related to the energy leakage of SAWs into the bulky air medium above the cavity array. Moreover, we find that by breaking the inversion symmetry (*h*_1_ ≠ *h*_2_), a gap forms between imaginary wavenumber parts for *q*^+^ and *p*^−^ modes at the K point. This means the attenuations of the modes with opposite pseudospins are different.

By gradually tuning the channel height *h*_c_, continuous evolutions of the Dirac point and the band edges at *q*^+^ and *p*^−^ points within a wide frequency range 0–5 kHz can be realized. Figure 1g shows that the frequencies for the Dirac, *q*^+^, and *p*^−^ points gradually increase with the increase of *h*_c_ and eventually approach constant values for SAWs. 3D pressure fields in the PCs with different channel heights 1, 10, and 50 mm are plotted in Fig. 1g (insets) for the *p*^−^ mode and in Supplementary Fig. 3 (insets) for the *q*^+^ mode. By comparing the pressure fields for different channel heights, it can be found that the interaction of acoustic waves with the ceiling becomes weaker as the ceiling height increases from 1 to 50 mm. When the channel height is very large (for example *h*_c_ ≥ 50 mm), most of the energy of the generated wave mode tends to cling to the bottom cavity array; additionally, the effect of the ceiling on the generated acoustic mode becomes negligible, as shown by the pressure field in Fig. 1g for *h*_c_ = 50 mm. In this scenario, the generated acoustic mode propagates along the bottom cavity array as spoof SAWs, whose energy can leak into the bulky space above the cavities. The evolutions of pressure fields and mode frequencies (Fig. 1g and Supplementary Fig. 3) reveal that WAWs confined in a waveguide between the top ceiling and the bottom cavity array can gradually become SAWs propagating along the bottom cavities, as the channel height gradually increases. Such transition allows the frequencies for the Dirac, *q*^+^, and *p*^−^ points to be continuously tuned down to nearly 0 kHz by reducing the ceiling height. The ability to continuously tune the frequency without changing the lattice constant would allow for adjusting the subwavelength ratio, i.e., the ratio between the lattice constant *a* and the wavelength *λ* of free space acoustic waves (Supplementary Fig. 4a). The parametric study also suggests that optimized hybrid channel-cavity PCs have the potential to support deep subwavelength manipulation (Supplementary Fig. 5). Valley Chern numbers obtained through the integration of Berry curvature are provided to evaluate the change of intervalley mixing induced by the decrease of the ceiling height (Supplementary Fig. 4b). The |*C*_K(K′)_| calculated through integration increases with the decrease of the ceiling height *h*_c_ from 50 to 0.01 mm, which means the Berry curvature becomes more localized at K (K′) points and the intervalley mixing becomes weaker.

The following subsections show that our PCs can support edge modes of both the WAWs and SAWs and realize unusual functions, including dispersion tuning of edge modes, space-frequency modulation of edge mode group velocities, and rainbow trapping of edge modes. Some more commonly studied functions of PCs are demonstrated in Supplementary Figs. 6–8 and Supplementary Note 1 and 2, including splitting waves, guiding waves at different frequencies along distinct paths, and steering waves through positive/negative refraction.

### Valley Hall edge states for WAWs and SAWs

**Fig. 2:**
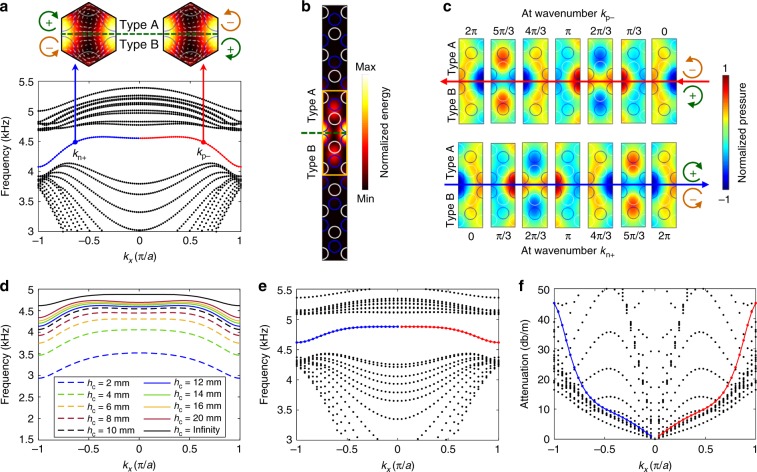
Acoustic valley Hall edge states and their dispersion curves. **a** Dispersion curves for the type A–B supercell, when the channel height is 10 mm and the deep and shallow cavity depths are 10 and 13 mm. The red and blue solid lines are dispersion curves for the edge mode with positive and negative wavenumbers. The black dotted lines are in the bulk bands. Inset: edge states at wavenumbers of *k*_p*−*_ and *k*_n+_. The color and arrows indicate acoustic energy and intensity, respectively. **b** Acoustic energy field in a supercell with an A–B interface, when the wavenumber is *k*_p*−*_ or *k*_n+_. **c** Evolution of acoustic pressure fields from 0 to 2π in the vicinity of the A–B interface. The top and bottom fields are for *k*_p*−*_ and *k*_n+_, respectively. The blue and white circles in **c** represent cavities in with depths of 10 and 13 mm. **d** Evolution of the edge mode dispersion with respect to channel height *h*_c_. With the increase of the channel height, the edge mode frequency for waveguide acoustic waves (WAWs) gradually increases and approaches to that for surface acoustic waves (SAWs). **e**, **f** Frequency and attenuation curves for the SAW-type edge mode, when the channel height is infinity. The attenuation is related to the leakage of SAW energy into the semi-infinite air medium above the cavities. The imaginary parts of wavenumbers for the SAW-type edge mode are plot in Supplementary Fig. 13.

**Fig. 3:**
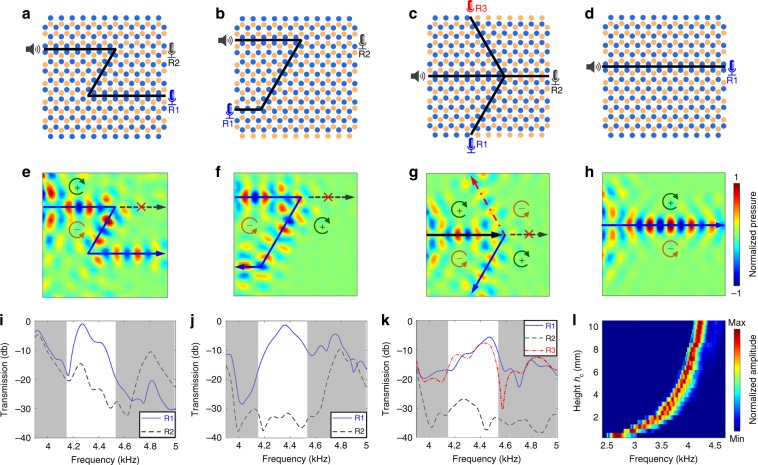
Experimental results for controlling the edge mode of waveguide acoustic waves. Valley phononic crystals with different interface configurations for **a** transporting acoustic waves along a Z-shaped path, **b** 180 degrees turning of acoustic waves, **c** dividing acoustic waves with a four-port topological junction, and **d** guiding acoustic waves along a straight path. The blue and brown circles represent cavities with depths of 10 and 13 mm, respectively. **e**–**h** Experimental pressure fields at 4.3 kHz for configurations in **a**–**d**, respectively. The circulation arrows represent directions of pseudospins. **i**–**k** Experimental transmission spectra for configurations in **a**–**c**, respectively. The shadow regions correspond to bulk bands. **l** Two-dimensional (2D) representation of normalized amplitudes versus frequency and channel height *h*_c_ for transmitted edge waves through the straight interface in **d**. The 2D representation shows that the frequencies of transmitted edge waves gradually increase from 2.6 to 4.3 kHz with the increase of channel height *h*_c_ from 0.4 to 10.4 mm.

A key feature of valley topological PCs, the valley Hall edge state^20,26^, can be achieved in hybrid channel-cavity PCs. Dispersion, attenuation, pseudospins, and temporal evolution of pressure fields for the edge mode are investigated through numerical simulations. The type A–B configuration (Fig. 2) with type A PCs above and type B PCs below the interface is selected as an example. The difference *∆h* between deep and shallow cavities is chosen to be 3 mm to provide a moderate level of symmetry breaking that does not introduce too strong intervalley mixing. From the derived dispersion relations (Fig. 2a) for *h*_c_ = 10 mm, a valley Hall edge mode (solid line) is observed in the frequency range 4.15–4.55 kHz. The energy field (Fig. 2b) at the wavenumber *k*_p*−*_ (or *k*_n*+*_) reveals that most energy is concentrated near the A–B interface, confirming that this is an edge mode. In the notations *k*_p*−*_ and *k*_n+_, the subscripts “p” and “n” are for positive and negative wavenumbers and “−” and “+” are for backward and forward-propagating waves, respectively. The acoustic intensity at the wavenumber *k*_p*−*_ features anticlockwise (−) and clockwise (+) pseudospins above and below the interface, respectively. At the wavenumber *k*_n+_, the pseudospin directions are reversed. Temporal evolutions of pressure fields for forward and backward propagating waves are given in Fig. 2c. As waves propagate backward with the wavenumber *k*_p−_, the pressure field evolution from 0 to 2π shows pseudospin− and pseudospin+ above and below the interface (Fig. 2c, top). In contrast, the pressure field evolution for forward-propagating waves shows the pseudospins reversed (Fig. 2c, bottom). To further characterize the edge mode transport, finite element simulations are performed (Supplementary Note 3). The simulation results (Supplementary Fig. 9) show that the pressure field in a plane at the top of the horizontal channel is nearly the same as that in a plane at the bottom of the channel. The generated edge mode is confined in the waveguide composed of a horizontal channel and an array of cavities; thus, the generated edge mode can be considered as a WAW-type edge mode. Such edge mode is demonstrated for multiple functionalities (see Supplementary Notes 4 and 5 for details). The results for robust routing, spin-locked wave division and frequency tuning of acoustic waves are shown Fig. 3 and Supplementary Fig. 10. The results for topological switches are provided in Supplementary Fig. 11.

The edge mode dispersion can be tuned by varying *h*_c_. As shown in Fig. 2d, the frequency of the WAW-type edge mode gradually increases with the increase of the channel height and gets closer to the edge mode frequency for PCs with *h*_c_ = infinity. To visualize the edge mode transport, numerical simulations are performed (Supplementary Note 6). The simulation pressure fields in Supplementary Fig. 12 show that an edge mode is guided along a Z-shaped interface. The simulation pressure in a plane near the bottom cavities is much larger than that in a plane 50 mm above. Moreover, the pressure amplitude drops rapidly with height. These observations indicate that an edge mode of SAWs, namely, “SAW-type edge mode”, propagates along the bottom cavity array and simultaneously is guided by the Z-shaped interface.

The dispersion relation for the SAW-type edge mode are derived, which involves complex wavenumbers. Figure 2e plots frequency as a function of the real part of the wavenumber (the imaginary part is given in Supplementary Fig. 13). The SAW-type edge mode has a nonzero imaginary wavenumber part *k*_imag_ for *k*_*x*_ ≠ 0 and |*k*_imag_| increases with the increase of |*k*_*x*_|. Figure 2f shows the edge mode attenuation rates, computed from |*k*_imag_|, increase as |*k*_*x*_| increases. We note the unexpected finding that some modes in bulk bands have lower attenuation rates than the edge mode.

### Robust routing and frequency tuning of SAW-type edge modes

**Fig. 4:**
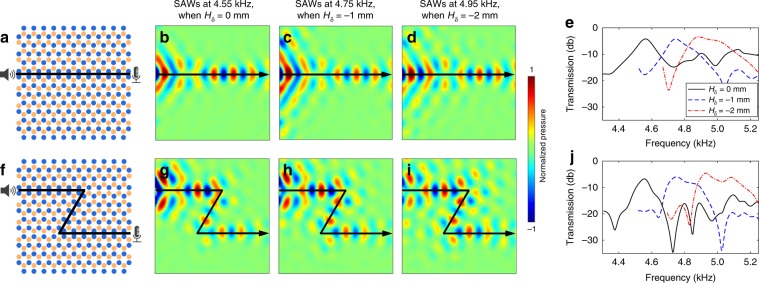
Experimental results for controlling the edge mode of surface acoustic waves. Configurations of valley phononic crystals (PCs) for transporting surface acoustic waves (SAWs) along **a** straight and **f** Z-shaped interfaces. The blue and brown circles represent shallow and deep cavities. Experimental pressure fields of SAWs propagating along the **b**–**d** straight and **g**–**i** Z-shaped paths at 4.55, 4.75, and 4.95 kHz, when the depth changes *H*_δ_ are 0, −1 and −2 mm, respectively. The acquired pressure fields confirm that the valley Hall edge mode of SAWs can be generated in our valley PCs. Experimental transmission spectra of SAWs along the **e** straight and **j** Z-shaped paths. As the depth change *H*_δ_ decreases, the edge mode’s frequency increases.

We experimentally prove the existence of SAW-type edge modes in our system with acoustic field measurements and demonstrate robust routing and frequency tuning of such modes. In experiments, we remove the top ceiling, and a speaker is placed near the bottom cavity array to generate incident waves. Two routes are demonstrated, one for transporting SAWs along a straight interface (Fig. 4a) and the other for guiding SAWs along a Z-shaped interface with two sharp corners (Fig. 4f). The pressure fields of SAW-type edge waves are acquired by scanning a microphone in a plane 10 mm away from the bottom cavity array. Figure 4b, g depict the obtained pressure fields for configurations with straight and Z-shaped interfaces, when the depths of shallow and deep cavities are *h*_sl_ = 10 mm and *h*_dp_ = 13 mm, respectively. The pressure fields clearly show that SAWs at 4.55 kHz can be transported along the two routes without obvious scattering even at sharp corners. We also quantitatively compare the experimental transmission spectra with simulation results in Supplementary Fig. 12j. Both the numerical and experimental spectra for receivers off the interface have transmissions lower than −15 db in the frequency range 4.51–4.88 kHz, while both spectra for receivers on the interface have transmissions above −15 db in the frequency range 4.51–4.65 kHz. The maximum experimental transmission is around *−*6.7 dB at 4.55 kHz, which is close to the maximum simulation transmission *−*3.6 dB at 4.68 kHz. The maximum transmission is smaller than 0 dB, as the SAW-type edge mode has an imaginary wavenumber part and gradually attenuates during the wave propagation along the interface. Note that the experimental spectrum for the receiver on the interface in Supplementary Fig. 12j has a dip at 4.72 kHz, whereas the dip in the simulation result is at 4.86 kHz. The slight frequency shift could be induced by errors in acoustic wave sensing experiments and inevitable fabrication errors of the actual sample.

In addition to the routing experiment at a frequency of 4.55 kHz (Fig. 4b, g), our acoustic system allows for tuning the dispersion of the SAW-type edge mode and thus efficiently transporting SAWs at other frequencies. To tune the frequency of the SAW-type edge mode, a depth change *H*_δ_ is applied on both the shallow and deep cavities. When *H*_δ_ is −1 mm, SAWs at an increased frequency of 4.75 kHz successfully transmit along the straight and Z-shaped interfaces (Fig. 4c, h). When the depth change *H*_δ_ further reduces to −2 mm, SAWs at a higher frequency of 4.95 kHz can be transported along the two interfaces. Transmission spectra for different depth changes *H*_δ_ are plotted in Fig. 4e for the straight interface and Fig. 4j for the Z-shaped interface. The spectra show that the frequencies for SAW-type edge waves increase as the cavity depths decrease. Moreover, the acquired pressure fields at the three frequencies of 4.55, 4.75, and 4.95 kHz show that the wavelengths at different frequencies are nearly the same. Simulation results of SAW-type edge waves at multiple frequencies (Supplementary Fig. 14) confirm the experimental observation. The fact that our valley PCs can be used to tune the frequency of the SAW-type edge mode while locking the wavenumber is an interesting feature of this system.

### Group velocity modulation and rainbow edge waves

Valley Hall edge modes at interfaces in valley PCs allow for robust transport, as has been observed in many acoustic systems^25–31^. Here we show that spatial tailoring of the edge mode dispersion allows for additional effects of interest. A straightforward reconfiguration of our acoustic system with a gradient in the cavity depths along the interface produces a chirped valley PC. Through theoretical, numerical, and experimental studies, we show that the edge mode dispersion curve and group velocity can be spatially modulated and that edge waves at different frequencies can be spatially separated with accumulated energy at different locations along the edge, manifested as the “acoustic rainbow” of the edge mode.

**Fig. 5:**
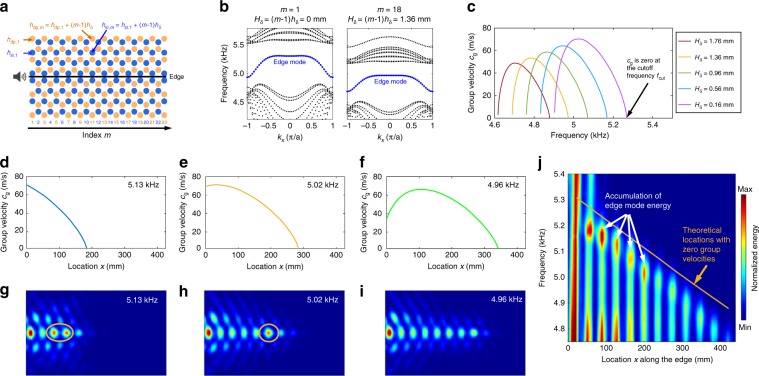
Demonstrations of rainbow edge waves in chirped valley phononic crystals. **a** Configuration of chirped valley phononic crystals (PCs) for transporting edge modes of surface acoustic waves (SAWs) along a straight interface. The blue and brown circles in the *m*^th^ column represent shallow and deep cavities with depths of *h*_sl*,m*_ and *h*_dp*,m*_. The cavity depths gradually increase with the increase of column index *m* following the relations *h*_sl*,m*_ = *h*_sl,1_ + (*m* − 1)*h*_δ_ and *h*_dp*,m*_ = *h*_dp,1_ + (*m* − 1)*h*_δ_. The depths of the shallow and deep cavities in the first column are *h*_sl,1_ = 8.58 mm and *h*_dp,1_ = 10.58 mm, respectively. The step size *h*_δ_ is 0.08 mm. **b** Wavenumber-frequency dispersion curves of edge modes for type A–B supercells with *H*_δ_ = 0 mm in the 1st column (left) and *H*_δ_ = 1.36 mm in the 18th column (right). The depth change *H*_δ_ equals to (*m* − 1)*h*_δ_. **c** Dispersion curves of edge mode group velocities *c*_g_ for type A–B supercells with different *H*_δ_ from 0.16 to 1.76 mm. The group velocity is zero at the cutoff frequency *f*_cut_. **d**–**f** Theoretical group velocities versus location *x* along the interface of the chirped valley PCs at frequencies of 5.13, 5.02, and 4.96 kHz. **g**–**i** Experimental energy fields of edge waves in the chirped valley PCs at frequencies of 5.13, 5.02, and 4.96 kHz. The energy fields show that SAWs at different frequencies stop propagating forward at different locations in the interface. Accumulation of edge mode energy in the low group velocity region can be observed in **g**, **h**. **j** Two-dimensional distribution of the edge mode energy with respect to location *x* along the interface and frequency. The locations where edge waves stop propagating forward agree well with the theoretically predicted locations with zero group velocity.

Figure 5a shows a schematic of a chirped valley PC composed of type A–B supercells with a horizontal straight edge (black solid line). The blue and brown circles represent locations of shallow and deep cavities. The depths of shallow and deep cavities in the *m*^th^ column are denoted as *h*_sl*,m*_ and *h*_dp*,m*_, which linearly increase with *m*, following the relations *h*_sl*,m*_ = *h*_sl,1_ + (*m* − 1)*h*_δ_ and *h*_dp*,m*_ = *h*_dp,1_ + (*m* − 1)*h*_δ_, where *h*_δ_ is the step size and (*m* − 1)*h*_δ_ is the depth change *H*_δ_. In our example, we take *h*_δ_ = 0.08 mm, *h*_sl,1_ = 8.24 mm, and *h*_dp,1_ = 11.24 mm. For the experiment of the SAW-type edge mode, the top ceiling is removed.

The variations of wavenumber-frequency and group velocity dispersion curves with respect to the column index *m* are investigated. Figure 5b presents the wavenumber-frequency dispersion relations for supercells in columns *m* = 1 and 18, showing an edge mode (blue dotted line) whose frequency decreases with *m*. Figure 5c compares group velocity *c*_g_ dispersion curves for different cavity depth changes *H*_δ_ from 0.16 to 1.76 mm. The edge mode shows a cutoff frequency *f*_cut_ at the trailing end of the group velocity curve. At the cutoff frequency, the group velocity becomes zero, and the edge mode is no longer supported above this frequency. As the depth change *H*_δ_ decreases, the maximum group velocity increases and the cutoff frequency *f*_cut_ shifts higher. It is also worth noting that the group velocities of the edge modes are much smaller than that for bulk acoustic waves in air (343 m/s), which means the edge mode can be used to effectively delay acoustic waves.

The chirped valley PCs, being composed of type A–B supercells with gradient dispersion relations, enable spatial modulation of the edge mode group velocity along a straight interface. Figure 5d–f present the dependence of the group velocity on location *x* long the interface at three selected frequencies of 5.13, 5.02, and 4.96 kHz, respectively. These group velocity plots have trailing edges where velocities gradually reduce in the +*x* direction and finally reach zero. As the frequency decreases, the location *x* with zero group velocity increases; waves can propagate further in the +*x* direction at a lower frequency.

Based on the spatial modulation of the edge mode’s group velocity in Fig. 5d–f, it is expected that edge waves at different frequencies will stop propagating forward at different locations and thus become spatially separated along a straight interface. This is confirmed by experiments performed using the configuration in Fig. 5a. The acquired energy fields of edge waves are given in Fig. 5g–i for the three selected frequencies used in Fig. 5d–f. The energy fields show that edge waves at different frequencies can be generated and confined in the interface of the chirped valley PCs, with the edge waves propagating further at lower frequencies. The locations where the experimental wave stops propagating (Fig. 5g–i) nicely match the locations where the computed group velocity goes to zero (Fig. 5d–f). Moreover, accumulations of edge mode energy (Fig. 5g, h) present in the low group velocity region. Simulations have been performed to confirm the experimental observations (Supplementary Note 7 and Supplementary Figs. 15–18). The accumulations of edge mode energy can be seen clearly from the simulation results in Supplementary Fig. 18. In addition to the spatial modulation of their group velocities, SAWs propagating in the chirped valley PCs are also subjected to continuous energy leakage during their propagation. As SAWs propagate further, their effectively transmitted energy will gradually decrease due to energy leakage. Therefore, using the SAW mode, it will be difficult to guide acoustic waves for a long distance with high transmission efficiencies. It will also be difficult to use chirped valley PCs for efficiently accumulating wave energy in low group velocity regions that are far from the wave source. Adverse effects caused by the energy leakage can be addressed by using WAWs in chirped valley PCs with a low ceiling height.

To further characterize the spatial modulation of edge waves, the wave energy distribution along the straight interface of the chirped valley PCs is acquired and plotted in Fig. 5j, a 2D representation of the wave energy distribution with respect to location *x* along the edge and frequency. The figure shows that the propagation distance of edge waves decreases as the frequency increases. The locations at which edge waves stop propagating forward agree well with the theoretically predicted locations with zero group velocity (brown solid line). We also note that as the frequency increases, the location with accumulated energy shifts to the left side of the interface. These features of rainbow edge waves can be seen more clearly from the 2D representation of simulation results in Supplementary Fig. 19. In our study, edge waves at different frequencies can be guided to distinct locations using the chirped valley PCs. This type of wave guidance is known as “rainbow guiding”^53^, which can be used to precisely control the wave guidance and may facilitate the design of acoustic circuits.

## Discussion

We have developed tunable valley PCs composed of hybrid channel-cavity cells with three tunable parameters and demonstrated that such PCs can realize multiple functionalities. Our experiments, supported by simulation results, show successful tuning of dispersion relations, robust routing of SAWs, control of wave attenuation, and modulation of edge mode group velocities. We have also shown that these PCs support acoustic topological states with complex wavenumbers and can be configured to form chirped valley PCs, which permit the formation of rainbow edge waves. These results demonstrate the value of this system for further scientific investigations and device development.

Although recently reported topological insulators^43^ and rotatable-unit-based valley PCs^34^ can realize reconfiguration of acoustic wave pathways, their functionalities rely heavily on simply changing the path configuration. Compared to those designs, our valley PCs enable more functionalities. Firstly, these PCs can tune multiple features of dispersion relations, including Dirac point frequencies, band-edge frequencies, band gaps, edge mode frequencies, and edge mode group velocities. Those mentioned frequencies can be tuned continuously over wide ranges while locking the wavenumber and phase pattern of acoustic waves; this feature could inspire the design of tunable wideband acoustic devices. Secondly, our PCs can adjust the imaginary parts of wavenumbers; thus, they can switch between decaying and nondecaying acoustic waves and control the wave amplitude and attenuation. Thirdly, they can continuously tune the subwavelength ratio, i.e., the ratio between the lattice constant *a* and the wavelength *λ* of free space acoustic waves (Supplementary Fig. 4); this ability could be useful for deep subwavelength manipulation.

There are intensive studies on topological systems with real wavenumbers^19–33^, but relatively few consider topological systems with complex wavenumbers. Using our PCs, we found that band crossing and Dirac cones for SAWs occur in a multidimensional space of complex wavenumber and frequency and that breaking the inversion symmetry opens a gap in the imaginary part of the wavenumber for *q*^+^ and *p*^−^ modes at the K point. This means the attenuation factors of modes with opposite pseudospins are different. Furthermore, we found that SAW-type edge modes have complex wavenumbers, implying that they decay exponentially as they propagate along an interface. Our work suggests possible benefits of the exploration of different types of topological systems with complex wavenumbers.

Our results on edge mode propagation in chirped valley PCs also suggest various possibilities. Our experiments and simulations confirm that edge modes can be generated in chirped valley PCs, and we demonstrated the space-frequency modulation of the edge mode group velocity as well as gradually slowing the group velocity to zero. We demonstrated rainbow edge waves where different frequency components of SAW-type edge modes are spatially separated with accumulated energy at different designated locations along the PCs’ interface. This could be useful for decomposition of frequency components in space, energy harvesting, and selective wave filtering.

Future developments will focus on miniaturizing the valley PCs for deep subwavelength manipulation and control of ultrasonic waves. The parametric study (Supplementary Fig. 5) suggests that the miniaturized PCs have the potential to support deep subwavelength manipulation with *a/λ* ~ 0.02. Moreover, by reducing both the lattice constant and cavity depth, miniaturized PCs could be developed for controlling ultrasonic waves. Such devices could be fabricated by advanced manufacturing techniques, such as replication-based forming^54–56^, high-resolution additive manufacturing^57–59^, and/or micromilling^60,61^. It should also be possible to reduce the response time of the system through automatic operation. To realize this, one potential method is to introduce automated pumps connected to the cavities. By controlling the automated pumps with a microcontroller board, we would expect to be able to control the effective cavity volumes with a response time would be similar to our previous result^41^ (30 s) for fully filling an empty cavity. For miniaturized devices, the response time would be further reduced. These research directions are important steps toward fast, miniaturized designs that can be integrated with precision electro-acoustic systems^62–64^ to develop advanced acoustic tweezers for high-resolution dynamic manipulation of nano- to micro-objects.

## Methods

### Fabrication and tuning of valley topological PCs

The valley topological PCs is composed of a 5 mm thick glass ceiling and a 25.4 mm thick acrylic plate containing cylindrical cavities distributed in 2D honeycomb lattices. The channel height (distance *h*_c_ between the glass ceiling and the acrylic plate) can be adjusted by changing the heights of four spacers placed at the four corners of the acrylic plate for supporting the glass ceiling. The cavities are fabricated through milling and have the same diameter of 8.7 mm and the same depth of 18 mm. The cavity centers are located at the vertices of a honeycomb with the distance *D* = 21.1 mm. The effective depth of each cavity can be tuned by pumping water into/out of the cavity with a syringe (Becton Dickinson, 5 ml Syringe) through a port connected to the bottom of the cavity or by directly pipetting water with a high precision pipette (Fisherbrand™ Elite Pipette with an error less than 2 µL). In our proof-of-concept design, the channel height and cavity volumes are manually controlled, but automated tuning using a microcontroller is clearly possible.

### Acquisition of acoustic fields

The acoustic field acquisition was performed in a 2D acoustic waveguide. Incident waves were generated by a loudspeaker with 0.9 cm radius. Acoustic pressures between the glass ceiling and the acrylic plate with cavities were acquired by a microphone attached on a 2D scanning system. Through point-by-point scanning, 2D acoustic pressure fields were acquired. At each scanning point, four pressure signals were acquired and then averaged to reduce the system noise.

### Derivation of valley Chern numbers

Through the **k**∙**p** perturbation method^20,65^, the valley Hall phase transition can be captured by the *∆h*-dependent continuum Hamiltonian $H_{\mathrm{K}}\left( {\delta {\mathbf{k}}} \right) = v_{\mathrm{D}}\left( {\delta k_x\sigma _x + \delta k_y\sigma _y} \right) + mv_{\mathrm{D}}^2\sigma _z$, where *v*_D_ is the Dirac velocity, *δ***k** is the momentum deviation **k**-**k**_K_ from the valley center K, and *σ*_*i*_ are Pauli matrices that operate on the vortex pseudospins. The effective mass *m* is $\left( {\omega _{{\mathrm{q + }}} - \omega _{{\mathrm{p - }}}} \right)/2v_{\mathrm{D}}^2$. The Hamiltonian depends on *∆h* through the frequencies *ω*_q*+*_ and *ω*_p−_ in the effective mass term. Using this Hamiltonian, the Berry curvature can be derived as $\Omega \left( {\delta {\mathbf{k}}} \right) = mv_{\mathrm{D}}\left( {|\delta {\mathbf{k}}|^2 + m^2v_{\mathrm{D}}^2} \right)^{ - 3/2}/2$. By further integrating the Berry curvature in the K valley, the associated valley Chern number can be obtained $C_{\mathrm{K}} = {\textstyle{\gamma \over {2{\uppi}}}} = {\textstyle{1 \over {2{\uppi}}}}{\int} {\Omega \left( {\delta {\mathbf{k}}} \right)} d^2\delta {\mathbf{k}}$, where *γ* is the Berry phase. The theoretical valley Chern numbers can be calculated using *C*_K_ _*=*_ sgn(*m*)/2 and *C*_K′_ = −sgn(*m*)/2 for K and K′ valleys, respectively^20^.

### Numerical simulations

The commercial finite element analysis software COMSOL Multiphysics is used for numerical simulations. The background medium is air with density and speed of sound being 1.2 kg/m^3^ and 343 m/s, respectively. The air-liquid interface is modeled by an impedance boundary with density and speed of sound in the liquid being 1000 kg/m^3^ and 1480 m/s, respectively. The glass ceiling and acrylic walls are modeled as rigid walls as their impedances are much larger than that of the background medium.

## Supplementary information

Supplementary Information

## Data Availability

Data supporting the findings of this study are available from the corresponding author upon reasonable request.
