# Supplementary material for: Dispersion tuning and route reconfiguration of acoustic waves in valley topological phononic crystals
Source: Nat Commun. 2020 Feb 7;11:762. doi: 10.1038/s41467-020-14553-0 (PMC7005747; doi:10.1038/s41467-020-14553-0)
Supplement: Supplementary file 1 — Supplementary Information [file 41467_2020_14553_MOESM1_ESM.pdf]

Supplementary Information for:

Dispersion Tuning and Route Reconfiguration of Acoustic Waves in Valley Topological Phononic Crystals

Tian and Shen et al.

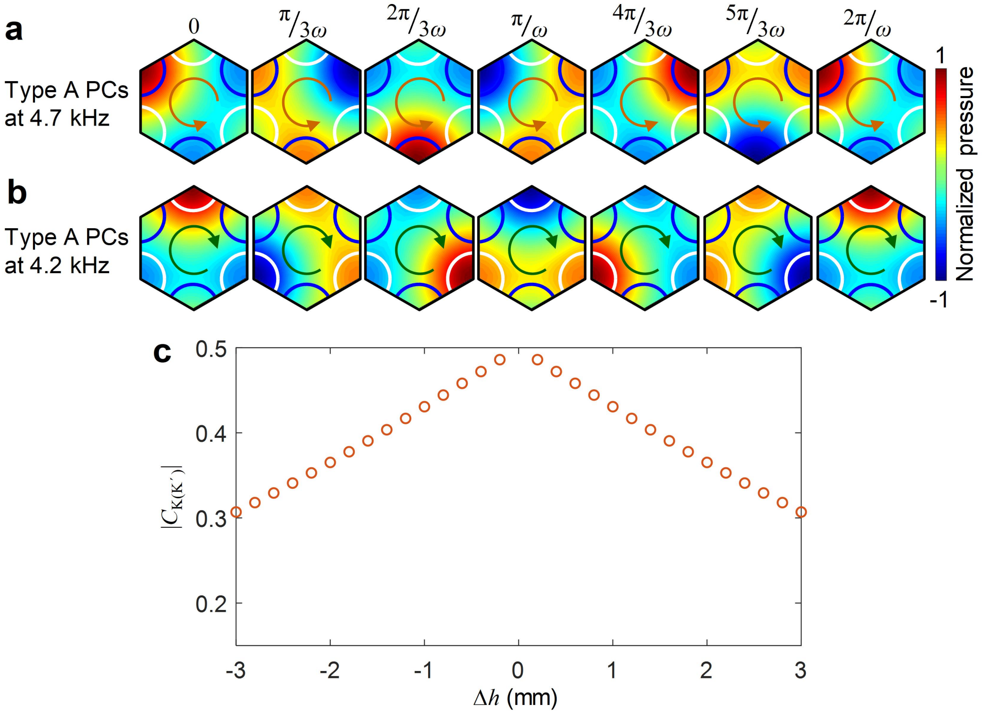

Supplementary Figure 1 | Temporal evolution of pressure fields in type A phononic crystals and calculated valley Chern numbers. (**a**) The 4.7 kHz pressure fields for the $p-$ mode in type A phononic crystals (PCs) from t = 0 to t = 2π/ω show pseudospin− around the spatial center of the eigenstate. (**b**) The 4.2 kHz pressure fields for the $q+$ mode in type A PCs from t = 0 to t = 2π/ω show pseudospin+ around the spatial center of the eigenstate. The blue and white arcs represent cavities with depths of 10.1 and 12.9 mm, respectively. The circular arrows represent directions of pseudospins. (**c**) Calculated absolute values of valley Chern number $|C_{K(K')}|$ with respect to cavity depth difference Δh. The valley Chern number is calculated by integrating the Berry curvature using the equation $C_K = \frac{\gamma}{2\pi} = \frac{1}{2\pi}\int \Omega(\delta\mathbf{k}) d^2\delta\mathbf{k}$, where $\delta\mathbf{k}$ equals to $\mathbf{k} - \mathbf{k}_K$ and γ is the Berry phase[1]. The Berry curvature $\Omega(\delta\mathbf{k})$ is $m v_D (|\delta\mathbf{k}|^2 + m^2 v_D^2)^{-3/2}/2$, in which m is the effective mass $(\omega_{q+} - \omega_{p-})/2v_D^2$ and v_D is the Dirac velocity[1]. $C_{K'}$ equals to $-C_K$. The integration is performed over a finite square around the K point with δk_x and δk_y in the range $(\frac{-\pi}{2a}, \frac{\pi}{2a})^2$. The

result $|C_{K(K')}|$ calculated through integration is smaller than the theoretical value ½ obtained with $|C_{K(K')}| = |\mathrm{sgn}(m)|$. This discrepancy is induced by the inversion symmetry breaking (i.e., $\Delta h \neq 0$). As the $|\Delta h|$ increases from 0.2 to 3 mm, the inversion symmetry breaking becomes stronger, and the calculated $|C_{K(K')}|$ decreases from 0.486 to 0.307. The decrease means the Berry curvature becomes less localized at K (K′) points and the intervalley mixing becomes stronger.

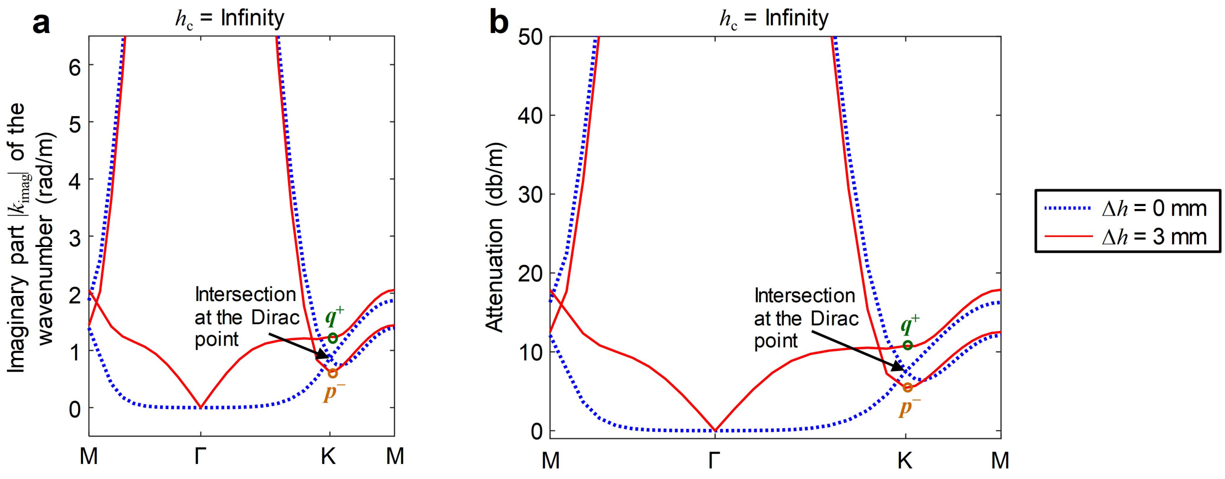

Supplementary Figure 2 | Complex wavenumber and attenuation curves for surface acoustic modes when the channel height is infinity. (**a**) Projection of the dispersion relation in the complex wavenumber space. The curves in the complex wavenumber space have an intersection at the Dirac point, when Δh = 0 mm (blue dotted lines). There is gap between the imaginary wavenumber parts for $q+$ and $p-$ modes at K, when Δh = 3 mm (red solid lines). (**b**) Attenuation curves with respect to the real wavenumber part. When Δh = 0 mm, attenuation curves have an intersection at the Dirac point (blue dotted lines). When Δh = 3 mm, there is a difference between attenuation values for the $q+$ and $p-$ modes at K (red solid lines). In addition, it can be found that the $p-$ mode has a lower attenuation at K, compared to the $q+$ mode. Note that the attenuation is caused by the leakage of the SAW energy into air in a semi- infinite space above the cavities of phononic crystals. The lattice constant a and cavity diameter d are 36.5 mm and 8.7 mm, respectively.

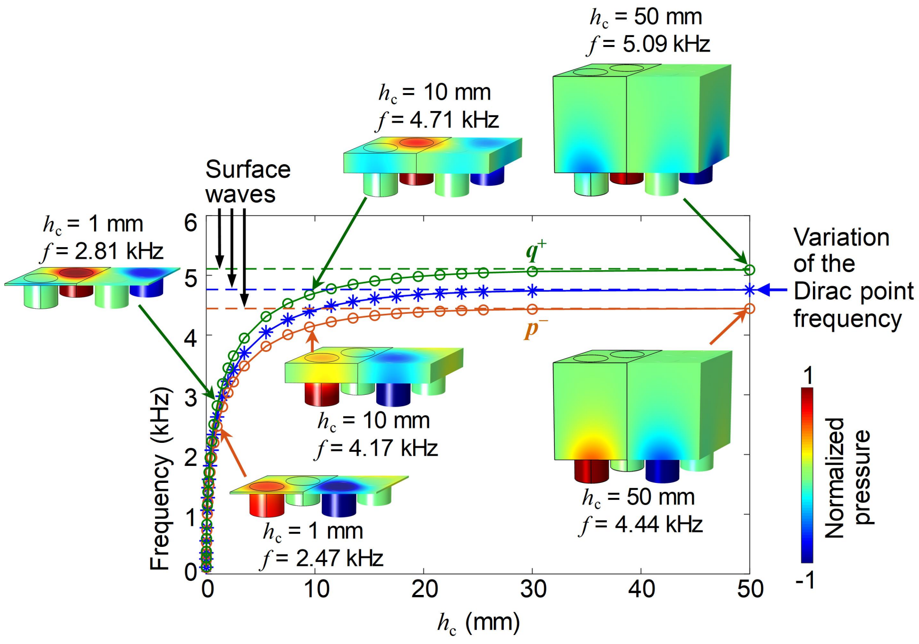

Supplementary Figure 3 | Dirac point and band edges frequencies at different channel heights. The results are obtained through finite element simulations. The lattice constant a and cavity diameter d are 36.5 mm and 8.7 mm, respectively. The depths of deep and shallow cavities are 13 and 10 mm, respectively. From the simulation results, it can be seen that the frequencies for Dirac, $q+$, and $p-$ points gradually increase and approach frequencies for surface acoustic modes, as the channel height increases. From the pressure fields for the $p-$ mode (insets), we can see that the deep cavities have larger pressure amplitudes than the shallow cavities. In contrast, the pressure fields for the $q+$ mode (insets) show that shallow cavities have larger pressure amplitudes than the deep cavities. When the channel height is small, acoustic waves can interact with the top ceiling. For this case, we consider the generated waves as waveguide acoustic waves (WAWs), since they propagate in a waveguide confined by a top ceiling and an array of cavities. When the channel height is large, acoustic waves cannot effectively interact with the top ceiling and acoustic pressures near the bottom cavities are

dominated. For this case, we consider the generated waves as surface acoustic waves (SAWs). In addition, evolutions of pressure fields as well as frequencies with respect to the channel height reveal that WAWs can gradually transform to SAWs as the channel height increases.

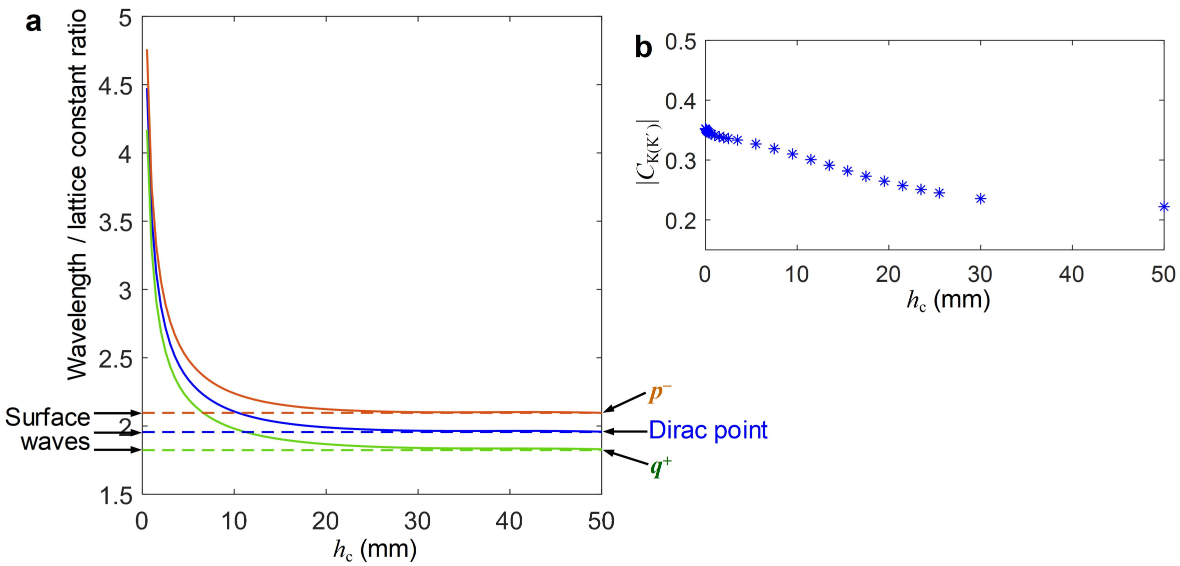

Supplementary Figure 4 | Continuous variation of the wavelength/lattice constant ratio with respect to the channel height. (**a**) The ratios (wavelength λ /lattice constant a) at q+, p− and Dirac points gradually increase with the decrease of the channel height h_c. This means by changing the channel height of our PCs, we can tune the λ/a ratio. The lattice constant is smaller than the wavelength of free space acoustic waves in air, which means our PCs can be used for subwavelength manipulation of acoustic waves. In our current experiment, the maximum λ/a ratio achieved is around 3.5. We believe that it is possible to further increase the λ/a ratio by optimizing our acoustic system as shown in Supplementary Figure 5, so that our system can be used for deep subwavelength manipulation. Note that for deriving these curves, only the channel height is changed; the lattice constant, cavity diameter, and cavities depths remain the same. (**b**) Calculated absolute values of valley Chern numbers $|C_{K(K')}|$ at different channel heights h_c. When the channel height h_c decreases from 50 to 0.01 mm, the cavity depth difference is kept at Δh = 3 mm for inversion symmetry breaking. The valley Chern number is calculated by integrating the Berry curvature over a finite square with δk_x and δk_y in the range $(\frac{-\pi}{2a}, \frac{\pi}{2a})^2$. The result $|C_{K(K')}|$

calculated through integration is smaller than theoretical value 1/2 obtained with $|C_{K(K')}|=|\mathrm{sgn}(m)|$, due to the inversion symmetry breaking (i.e., Δh = 3 mm). The calculated $|C_{K(K')}|$ increases with the decrease of h_c from 50 to 0.01 mm, which means the Berry curvature becomes more localized at K (K′) points and the intervalley mixing becomes weaker. In addition, from the equations for the valley Chern number and Berry curvature (see Methods), it can be found that the increase of $|C_{K(K')}|$ could be related to the decrease of the band gap that has been found in Supplementary Figure 3.

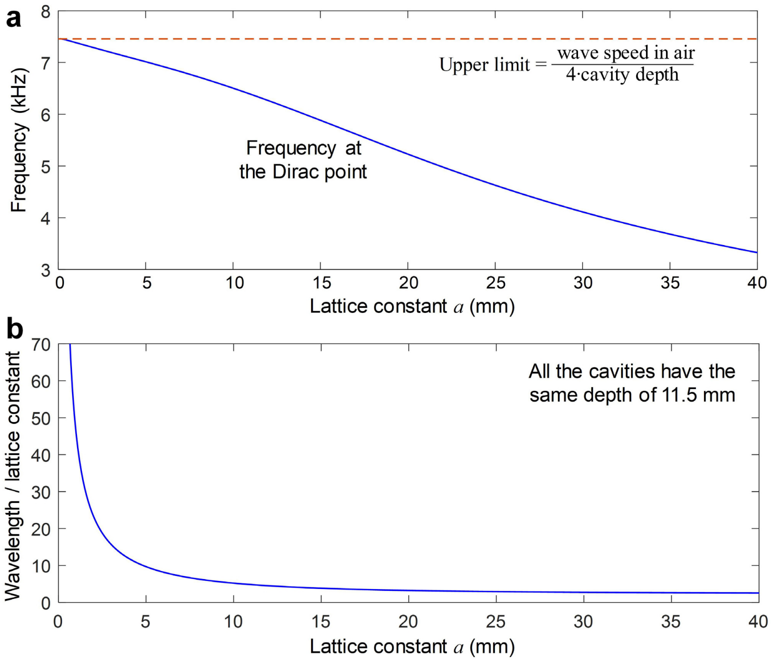

Supplementary Figure 5 | Optimization of the phononic crystals for deep subwavelength manipulation. To calculate these curves, we keep the channel height at 3 mm and the cavity depths at h_1= h_2=11.5 mm, and gradually scale up/down the original phononic crystals (PCs) composed of hybrid channel-cavity cells. For different lattice constants *a*, the frequencies at Dirac points and the corresponding wavelength/lattice constant ratios λ/a are calculated. (**a**) Frequency at the Dirac point with respect to the lattice constant *a*. An interesting finding is that as the lattice constant gradually decreases to 0 mm, the frequency at the Dirac point gradually increases and approaches an upper limit of 7.46 kHz. A reason for this trend is that the upper frequency limit depends on the cavity depth h_1, as given by the equation $0.25c_{air}/h_1$. Since the upper frequency limit is determined by the cavity depth, we can potentially use the hybrid channel-cavity PCs with small cavity depths for manipulating ultrasonic waves. (**b**) Wavelength/lattice constant ratio λ/a with respect to the lattice constant *a*. As the lattice constant gradually decreases, the ratio λ/a greatly increases, which offers the potential to use to hybrid channel-cavity PCs for deep subwavelength manipulation.

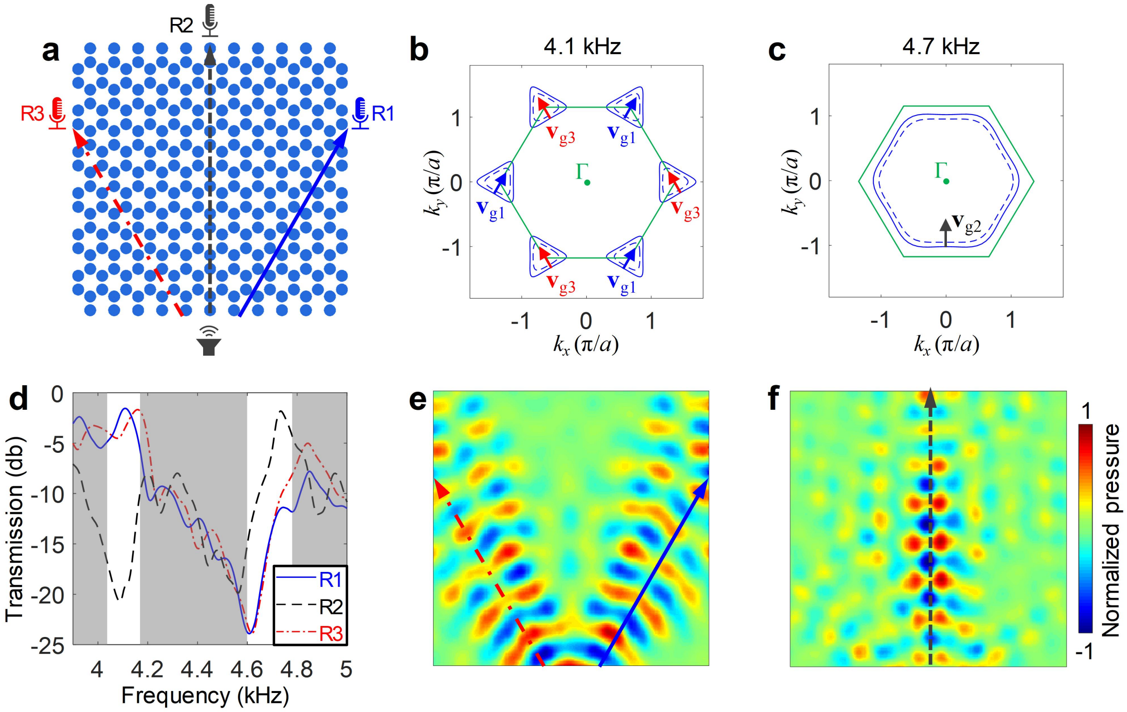

Supplementary Figure 6 | Experimental demonstrations of frequency-dependent acoustic routing for waves coming from the lower side of the phononic crystals. (**a**) Configuration of the phononic crystals (PCs) with $h_1 = h_2 = 11.5$ mm. In (**b**) and (**c**), the solid curves are equifrequency contours (EFCs) at 4.1 and 4.7 kHz, the dashed curves are EFCs at slightly higher frequencies, and the green hexagons indicate the first Brillouin zone (FBZ). (**d**) Transmission coefficients at the three receivers R1-R3. The white stripes illustrate frequency bands for wave splitting and confinement. (**e**) and (**f**) Experimental pressure fields for frequency-dependent acoustic routing when the lower side of the PCs is excited by a point source. At 4.1 kHz, acoustic waves are split into two branches, while at 4.7 kHz, the acoustic waves are guided along the central branch.

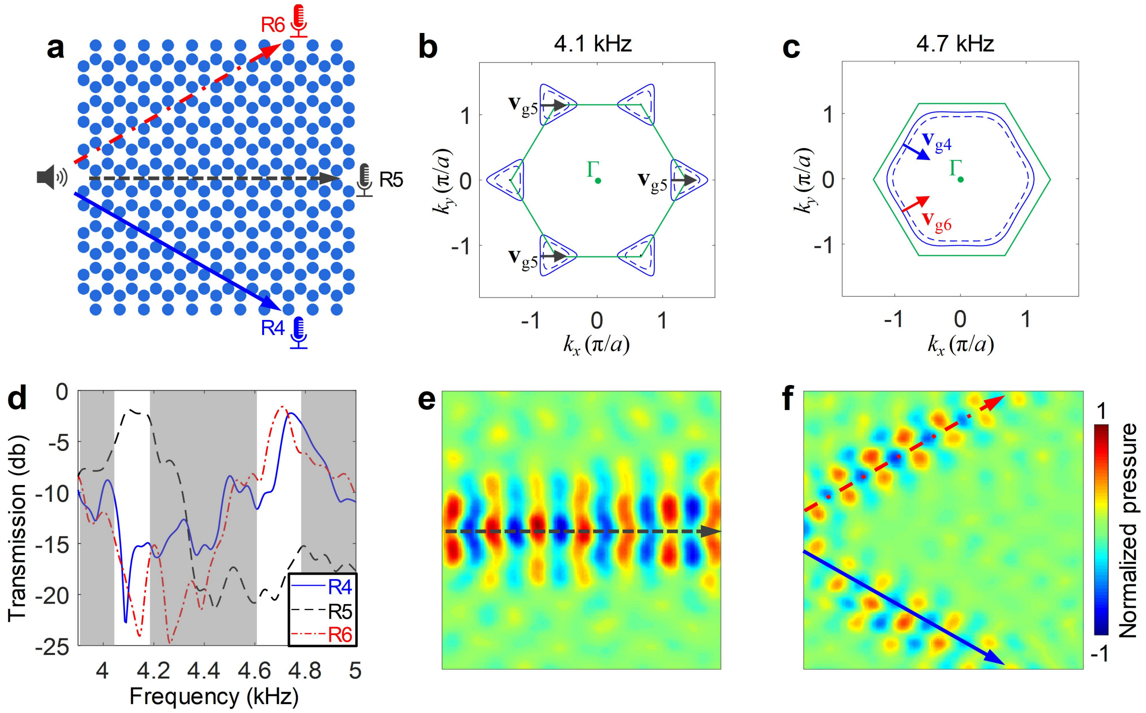

Supplementary Figure 7 | Experimental demonstrations of frequency-dependent acoustic routing for waves coming from the left side of the phononic crystals. (**a**) Configuration of the phononic crystals (PCs) with $h_1 = h_2 = 11.5$ mm. In (**b**) and (**c**), the solid curves are equifrequency contours (EFCs) at 4.1 and 4.7 kHz, the dashed curves are EFCs at slightly higher frequencies, and the green hexagons indicate the first Brillouin zone (FBZ). (**d**) Transmission coefficients at the three receivers R4-R6. (**e**) and (**f**) Experimental pressure fields for frequency-dependent acoustic routing when the left side of the PCs is excited by a point source. The acoustic waves are guided along the central branch at 4.1 kHz and split into two branches at 4.7 kHz.

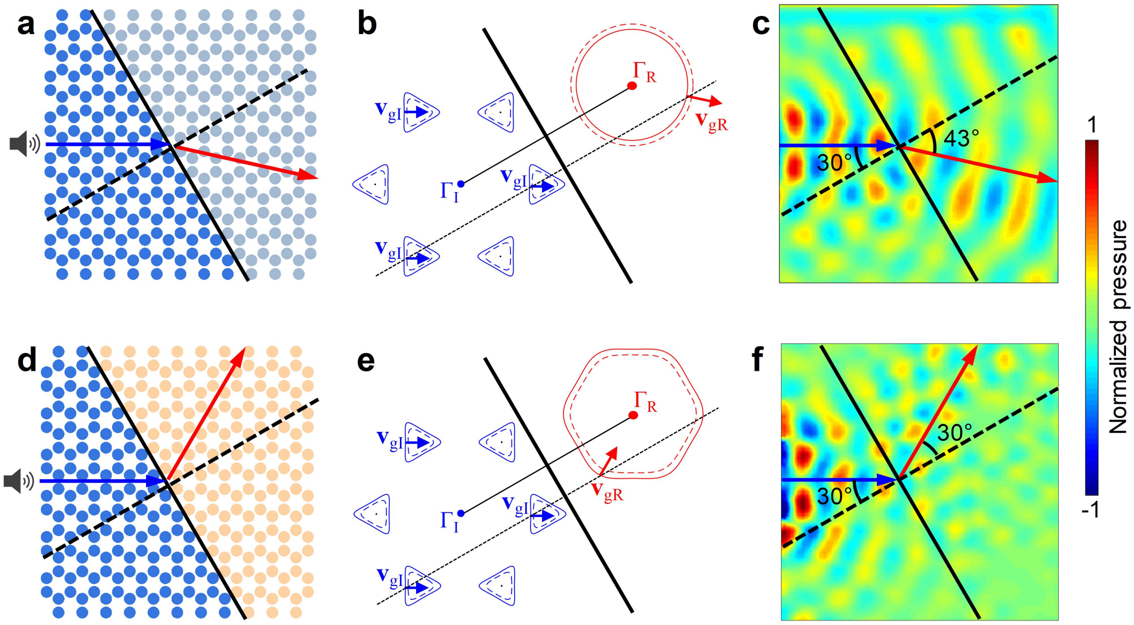

Supplementary Figure 8 | Experimental demonstrations of positive and negative refraction. (**a**-**c**) Configuration, equifrequency contour (EFC) analysis, acquired acoustic pressure field at 4.3 kHz for positive refraction between phononic crystals (PCs) with cavity depths of 10.5 mm (left side of the interface) and 1 mm (right side of the interface). (**d**-**f**) Configuration, EFC analysis, acquired acoustic pressure field at 4.3 kHz for negative refraction between PCs with cavity depths of 10.5 mm (left side of the interface) and 13.5 mm (right side of the interface). The solid curves in (**b**) and (**e**) are EFCs at the operating frequency and the dashed curves are EFCs at a slightly higher frequency. The blue and red arrows indicate directions of incident and refracted waves. The tilted black solid lines represent the interface between two types of PCs.

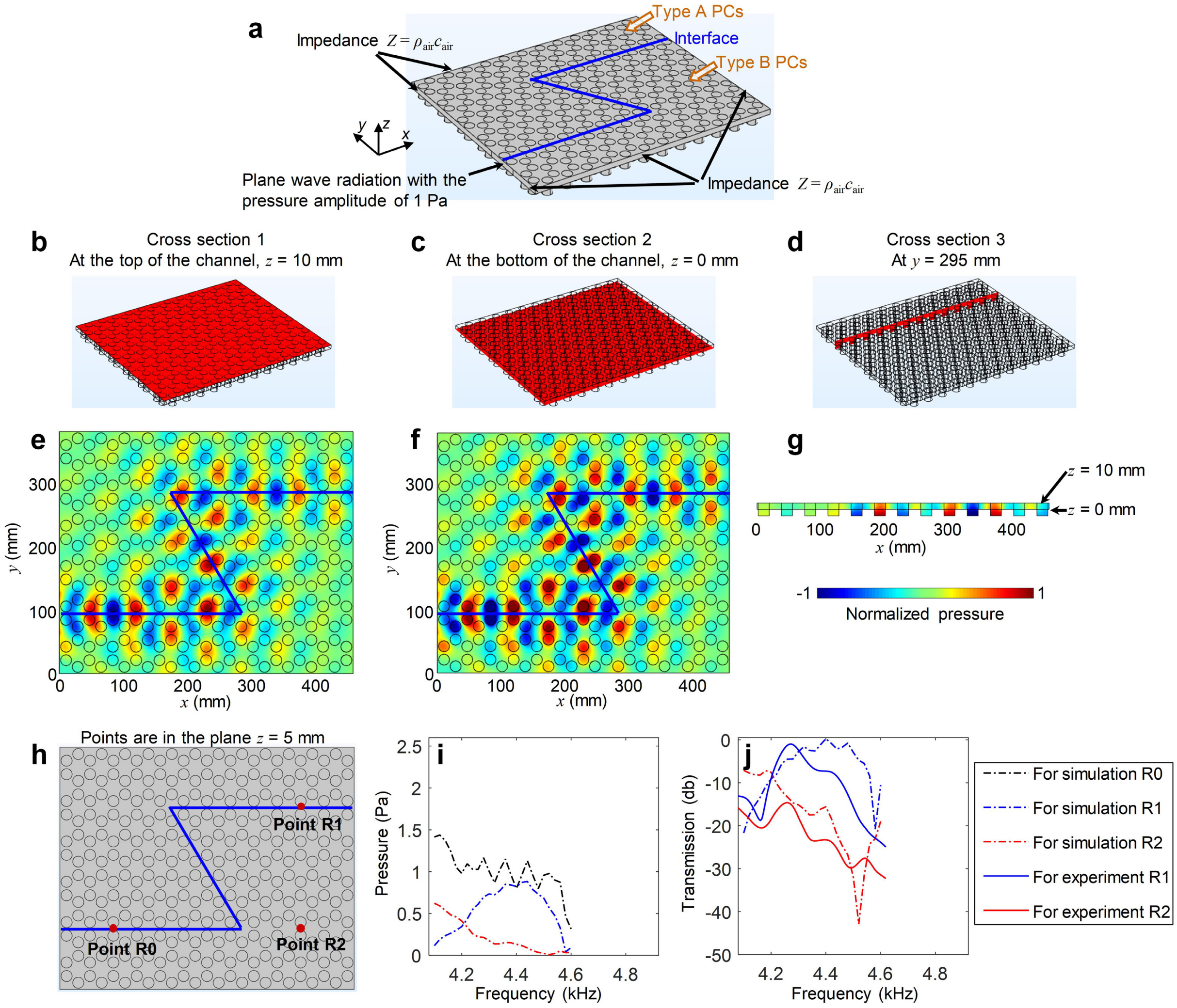

Supplementary Figure 9 | Simulation results at 4.3 kHz for demonstrating the valley Hall edge mode of waveguide acoustic waves. (**a**) Schematic of the simulation setup. The channel height h_c is 10 mm. The lattice constant a and cavity diameter d are 36.5 and 8.7 mm, respectively. The depths of deep and shallow cavities are 12.5 and 10.5 mm, respectively. The blue line represents the interface between type A and B phononic crystals (PCs). (**b-d**) Schematics for illustrating cross section 1 at z = 10 mm the top of the channel, cross section 2 at z = 0 mm the bottom of the channel, and cross section 3 at y = 295 mm (red areas). (**e-g**) Simulation pressure fields at 4.3 kHz for cross section 1, 2 and 3, respectively. The pressure

fields show that a valley Hall edge mode propagates along the Z-shaped interface. The generated edge waves can be considered as waveguide acoustic waves (WAWs) that are confined in a waveguide composed of a horizontal channel and an array of cavities. The edge mode is referred to as a 'WAW-type edge mode'. (**h**) Schematic to illustrate the relative positions of the sensing points R0 (it is on the interface and close to the entrance of the Z-shaped path), R1 (it is on the interface and close to the exit of the Z-shaped path), and R2 (it is off the interface and has the same x coordinate as R1). (**i**) Simulation pressure amplitudes obtained at R0-R2. (**j**) Quantitative comparison of transmission spectra obtained through simulations and experiments. At the receiver R2 (off the interface), both the numerical and experimental results have transmissions lower than −10 db in 4.22 ~ 4.60 kHz. At the receiver R1 (on the interface), both the numerical and experimental results have transmissions above −10 db in 4.22 ~ 4.46 kHz. The maximum experimental transmission is around −1 db at 4.27 kHz, which is close to the maximum simulation transmission 0 db at 4.40 kHz.

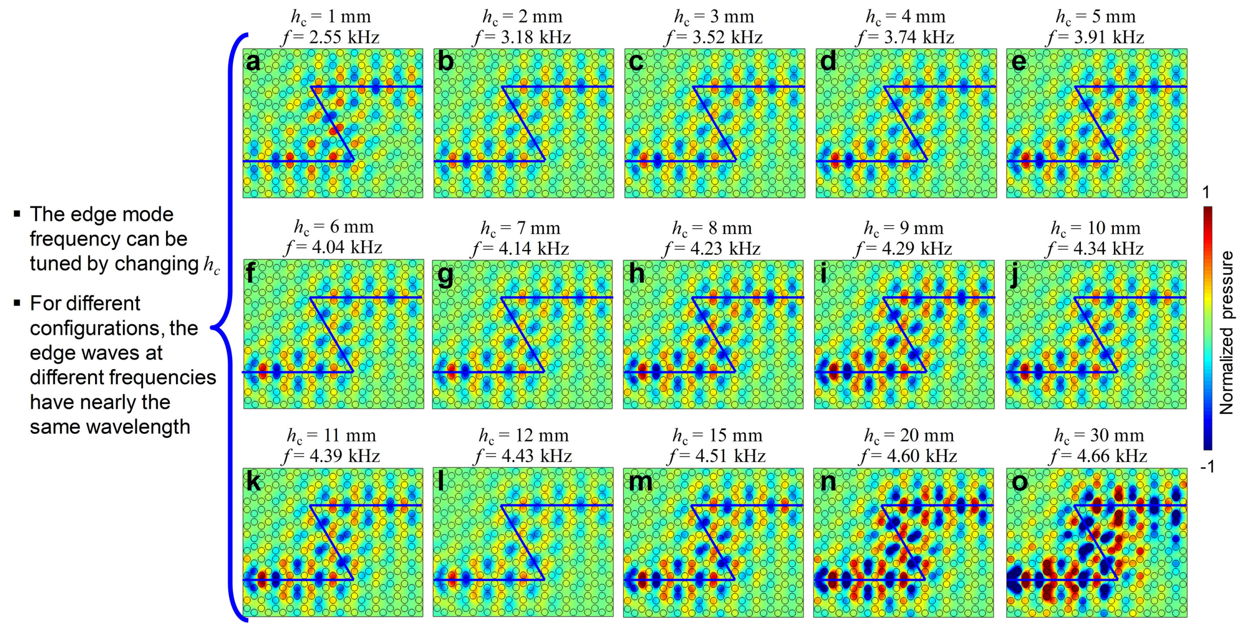

Supplementary Figure 10 | Simulation pressure fields for demonstrating the frequency tuning of the edge mode of waveguide acoustic waves by changing the channel height. (a-o) Simulation pressure fields at multiple frequencies from 2.55 to 4.66 kHz for different channel heights h_c from 1 to 30 mm. The edge mode of waveguide acoustic waves can be generated and guided along Z-shaped paths at different frequencies. The phase patterns for all the edge waves are nearly identical. This means for different channel heights the generated edge waves at different frequencies have nearly the same wavelength. Moreover, the edge mode frequency gradually increases with the increase of the channel height h_c.

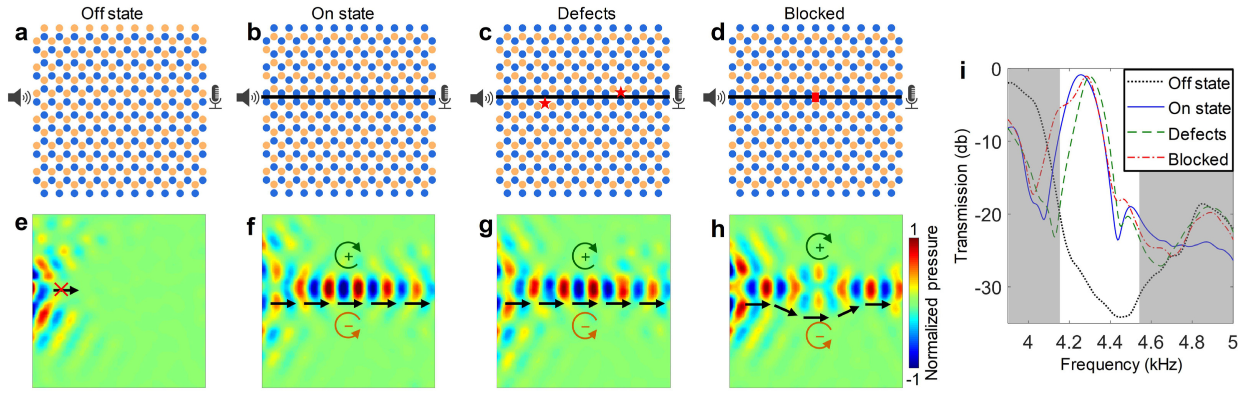

Supplementary Figure 11 | Experimental demonstrations of a topological switch for acoustic waves based on the acoustic valley Hall edge mode. Configurations of the switch at (**a**) off and (**b**) on states without and with a straight interface. Configurations for interfaces with (**b**) no defect, (**c**) two defects, and (**d**) an obstacle. The red stars in (**c**) are at two defects, which are full-fluid cavities. The red rectangle in (**d**) represents the obstacle, which is a scatterer made of an acrylic block (in-plane size 15 mm by 20 mm, height 10 mm). The blue and brown circles represent cavities with depths of 10 and 13 mm. (**e-h**) Experimental pressure fields at 4.3 kHz for configurations in (**a-d**), respectively. (**i**) Experimental transmission spectra for the four configurations in (**a-d**). The shadow regions correspond to the bulk bands. In (**f-h**), the circular arrows represent directions of pseudospins.

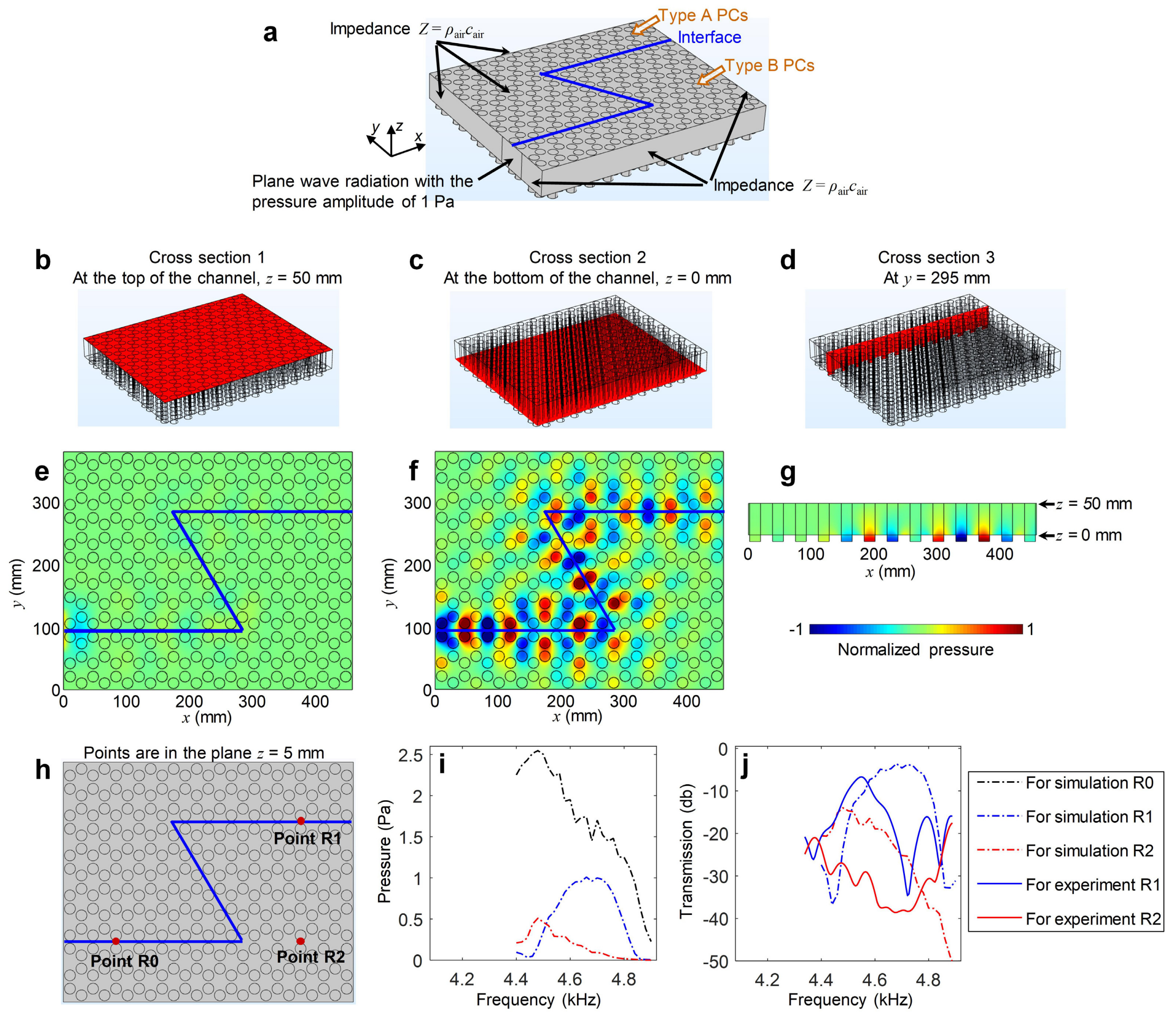

Supplementary Figure 12 | Simulation results at 4.65 kHz for demonstrating the valley Hall edge mode of surface acoustic waves. (**a**) A schematic of the simulation setup. The lattice constant a and cavity diameter d are 36.5 and 8.7 mm, respectively. The depths of deep and shallow cavities are 12.5 and 10.5 mm, respectively. The blue line represents the interface between type A and B phononic crystals (PCs). (**b-d**) Schematics for illustrating cross section 1 at z = 50 mm the top of the channel, cross section 2 at z = 0 mm the bottom of the channel, and cross section 3 at y = 295 mm (red areas). (**e-g**) Simulation pressure fields at 4.65 kHz for cross section 1, 2, and 3, respectively. The pressure fields show that a valley Hall edge mode of SAWs

is excited. The edge mode exhibits high energy near the bottom cavities and propagates along the *Z*-shaped interface, which is referred to as a 'SAW-type edge mode'. (**h**) Schematic to illustrate the relative positions of the sensing points R0 (on the interface and close to the entrance of the Z-shaped path), R1 (on the interface and close to the exit of the Z-shaped path), and R2 (off the interface and having the same x coordinate as R1). (**i**) Simulation pressure amplitudes obtained at R0-R2. (**j**) Quantitative comparison of transmission spectra obtained through simulations and experiments. At R2 (off the interface), the numerical and experimental results have transmissions lower than −15 db in 4.51 ~ 4.88 kHz. At R1 (on the interface), both results have transmissions above −15 db in 4.51 ~ 4.65 kHz. The maximum experimental transmission at R1 is around −6.7 db at 4.55 kHz, which is close to the maximum simulation transmission −3.6 db at 4.68 kHz. Note that the experimental result for R1 has a dip at 4.72 kHz, while the dip in the simulation result is at 4.86 kHz. This discrepancy could be induced by the errors in acoustic wave sensing experiments and slight geometrical errors between the experimental PCs and the simulation model.

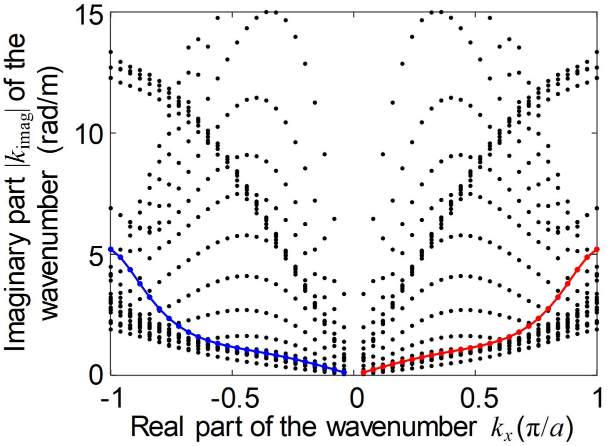

Supplementary Figure 13 | Imaginary parts of wavenumbers for the edge mode of surface acoustic waves in the type A-B supercell. The blue and red curves are for the edge mode with negative and positive real parts k_x of wavenumbers.

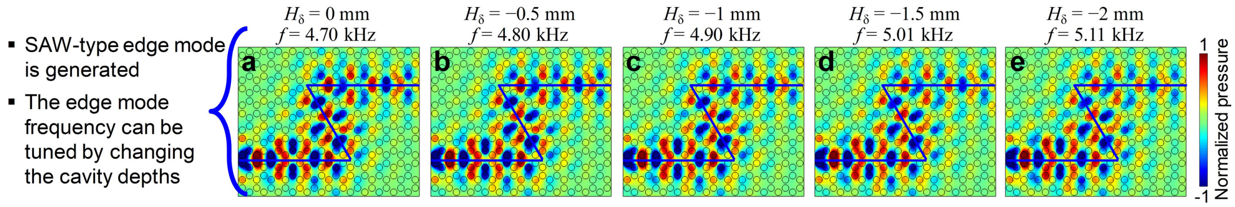

Supplementary Figure 14 | Simulation pressure fields for demonstrating the frequency tuning of the edge mode of surface acoustic waves by changing the cavity depths. Simulation pressure fields for (**a**) depth change H_δ = 0 mm at 4.70 kHz, (**b**) depth change H_δ = −0.5 mm at 4.80 kHz, (**c**) depth change H_δ = −1 mm at 4.90 kHz, (**d**) depth change H_δ = −1.5 mm at 5.01 kHz, and (**e**) depth change H_δ = −2 mm at 5.11 kHz. Note that in our simulations the same depth change H_δ is applied on both the shallow and deep cavities, and their depths become $h_{sl} + H_\delta$ and $h_{dp} + H_\delta$. The edge mode of surface acoustic waves (SAWs) can be generated and guided along a Z-shaped path at different frequencies. The phase patterns for all the edge waves are nearly identical, which means edge waves at those different frequencies have nearly the same wavelength. Moreover, the frequency of the SAW-type edge mode gradually increases with the decrease of cavity depths.

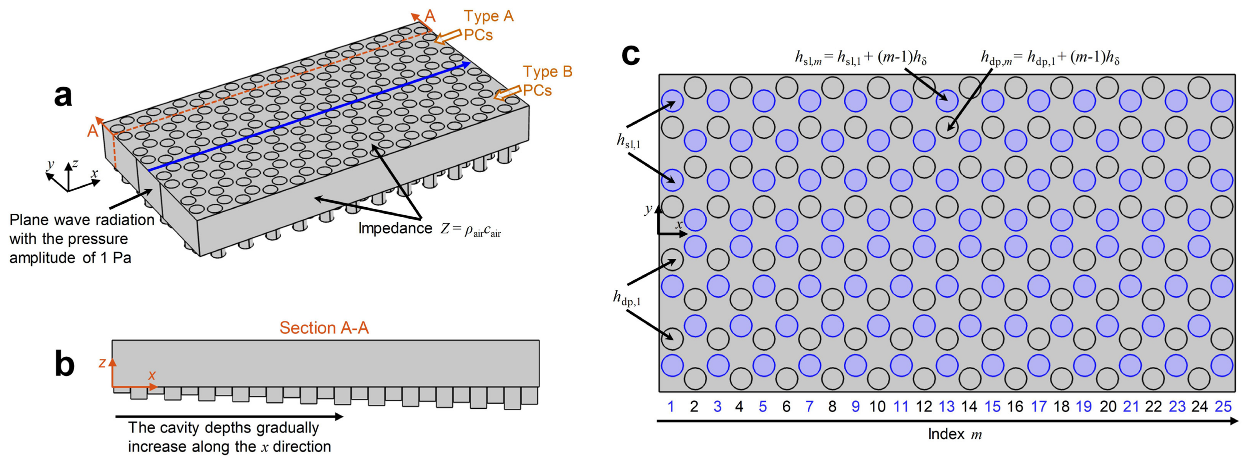

Supplementary Figure 15 | Schematics of the simulation setup for investigating rainbow edge waves in chirped valley phononic crystals. (**a**) Three-dimensional (3D) schematic of the simulation setup. The chirped valley phononic crystals (PCs) contain A-B type supercells with a straight interface (blue line). (**b**) Section view of the model from the plane A-A to the $+y$ direction. The section view shows that the cavity depths in supercells gradually increase along the x direction. (**c**) Top view of the model. The blue and grey circles in the mth column illustrate shallow and deep cavities with depths of $h_{sl,m}$ and $h_{dp,m}$. In our model, the lattice constant a and cavity diameter d are 36.5 and 8.7 mm, respectively. The depths of the shallow and deep cavities in the first column are $h_{sl,1}$ = 8.58 mm and $h_{dp,1}$ = 10.58 mm, respectively. With the increase of the column index m, the cavity depths linearly increase with a step size of h_δ = 0.08 mm. The simulation is performed in COMSOL Multiphysics. The plane wave radiation condition is applied on a surface at the left end of the interface. Matched impedance boundary conditions are applied on the top and side boundaries of the channel with the impedance Z being $\rho_{air}c_{air}$, where ρ_{air} and c_{air} are the air density and sound speed. Since the applied impedance matches with that of the background air, the effective height of the channel can be considered as infinity. The rest boundaries of the valley PCs are set as hard walls.

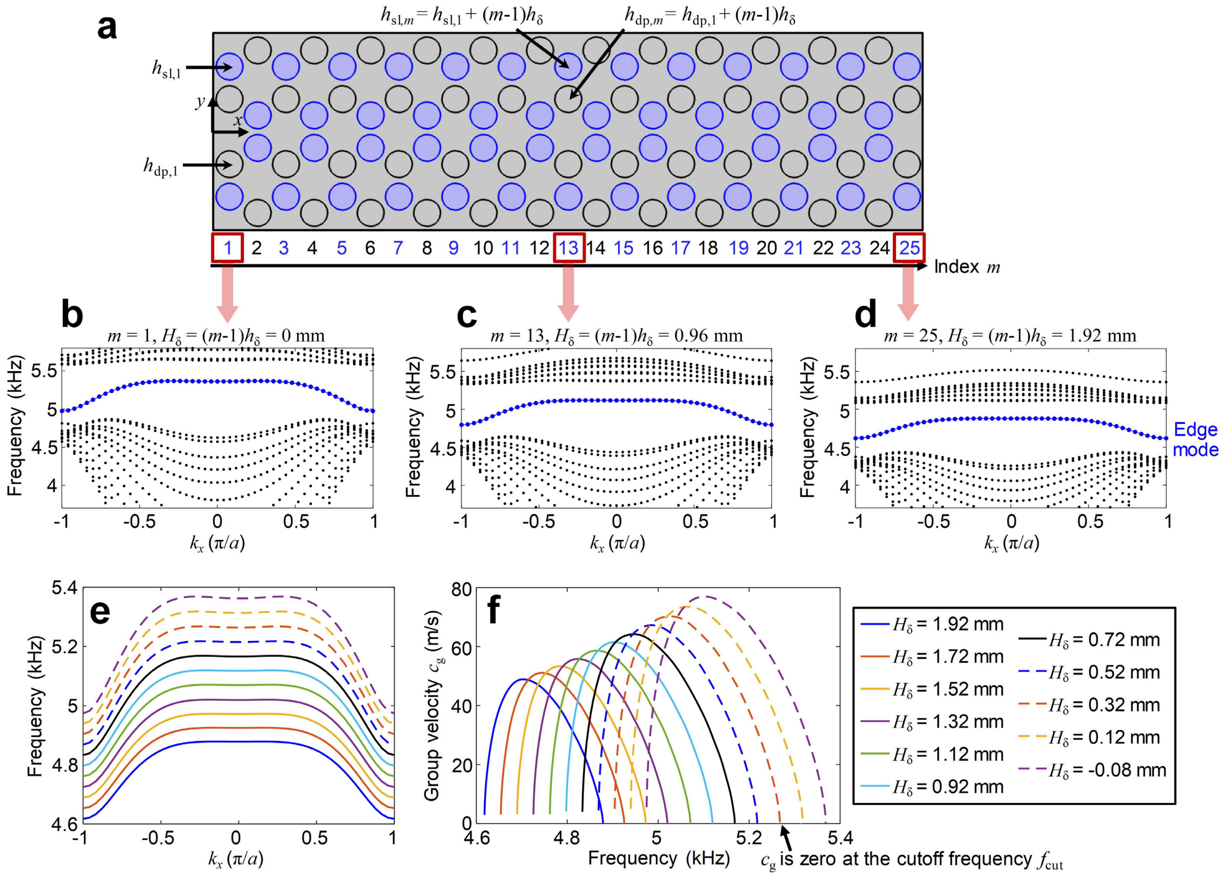

Supplementary Figure 16 | Variations of edge mode dispersion curves in chirped valley phononic crystals. (**a**) A schematic of the chirped valley phononic crystals (PCs), which contain A-B type supercells with a straight interface. The depths $h_{sl,m}$ and $h_{dp,m}$ for shallow and deep cavities linearly increase with the increase of the column index m. The depths $h_{sl,1}$ and $h_{dp,1}$ for the first column are 8.58 and 10.58 mm, respectively. The step size h_δ is 0.08 mm. (**b-d**) Wavenumber-frequency dispersion relations for A-B type supercells in the 1st, 13th, and 25th columns of the chirped valley PCs. (**e**) Wavenumber-frequency dispersion curves for different cavity depth changes H_δ from −0.08 to 1.92 mm. The edge mode frequency gradually decreases with the increase of the depth change H_δ. (**f**) Group velocity dispersion curves for different cavity depth changes H_δ from −0.08 to 1.92 mm. With the decease of the depth change H_δ, the

maximum group velocity becomes larger and the cutoff frequency f_{cut} increases. Moreover, the group velocities are much smaller than the wave speed 343 m/s in air, which means the edge mode can be used to effectively delay acoustic waves.

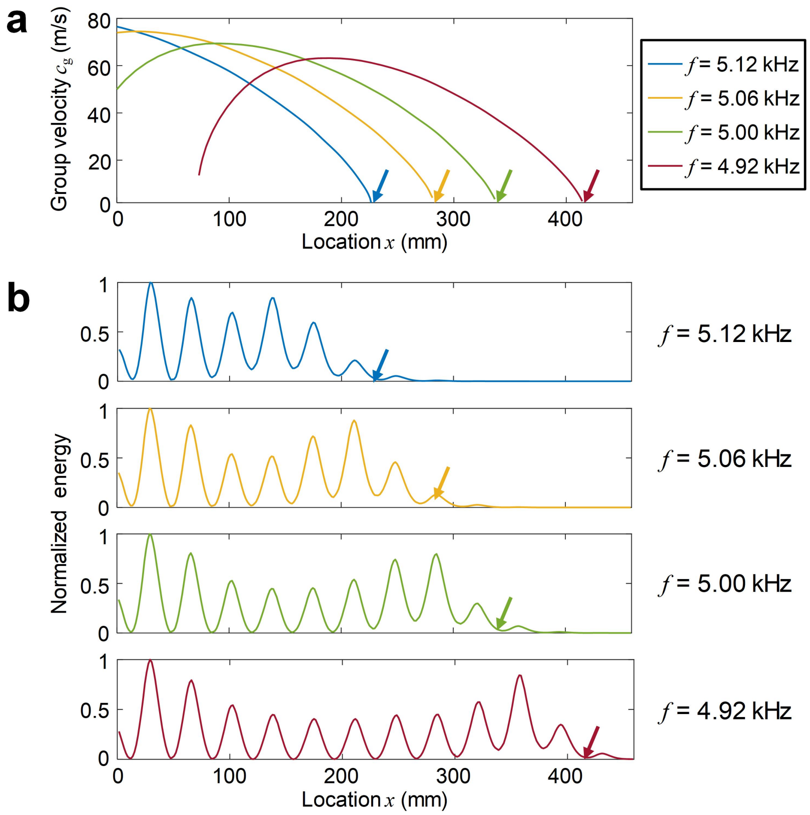

Supplementary Figure 17 | Variations of group velocities and energy of edge waves along the interface of the chirped valley phononic crystals. (**a**) Calculated group velocities versus the location *x* in the chirped valley phononic crystals (PCs) at frequencies of 4.92, 5.00, 5.06, and 5.12 kHz. Group velocities gradually slow down along the +*x* direction and finally reach zero at the locations marked with colored arrows. As the frequency decreases, the *x* location with the zero group velocity increases, which means waves can propagate further along the +*x* direction at a lower frequency. (**b**) Normalized wave energy distributions extracted from the simulation results along the interface of the chirped valley PCs. The energy distributions show that waves at different frequencies stop propagating forward at different *x* locations, which match with the locations with zero group velocity marked with colored arrows in (**a**). Therefore, we can use the group velocity curve in (**a**) to predict the locations, at which waves stop propagating forward.

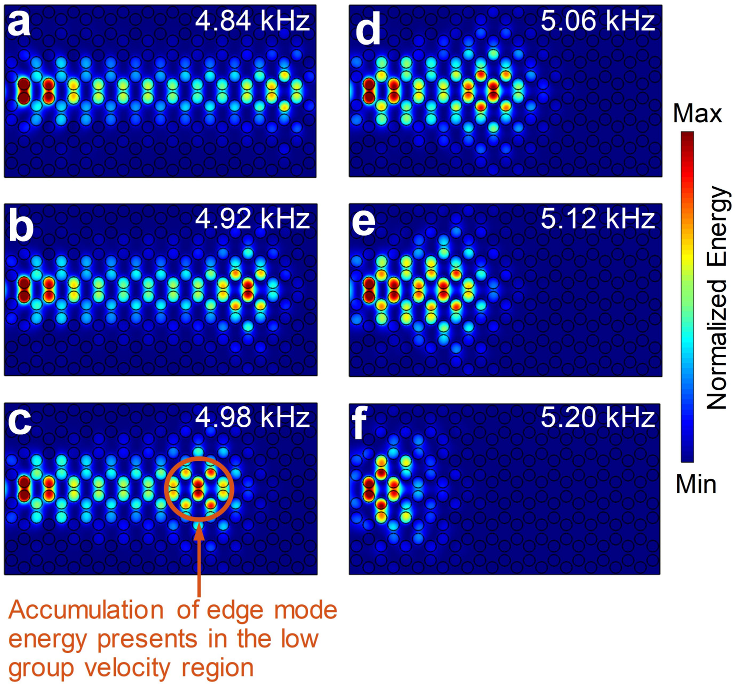

Supplementary Figure 18 | Simulation energy fields of edge waves in chirped valley phononic crystals. (**a-f**) Normalized wave energy distributions in the plane $z = 1$ mm (i.e., 1 mm above the cavities) of the chirped valley phononic crystals (PCs) at frequencies of 4.84, 4.92, 4.98, 5.06, 5.12, and 5.20 kHz. The energy distributions show that the edge mode of surface acoustic waves can be generated and confined in the edge of the chirped valley PCs at different frequencies. As the frequency decreases, edge waves propagate further. Moreover, the wave energy is accumulated and localized at different positions, which correspond to the low group velocity regions.

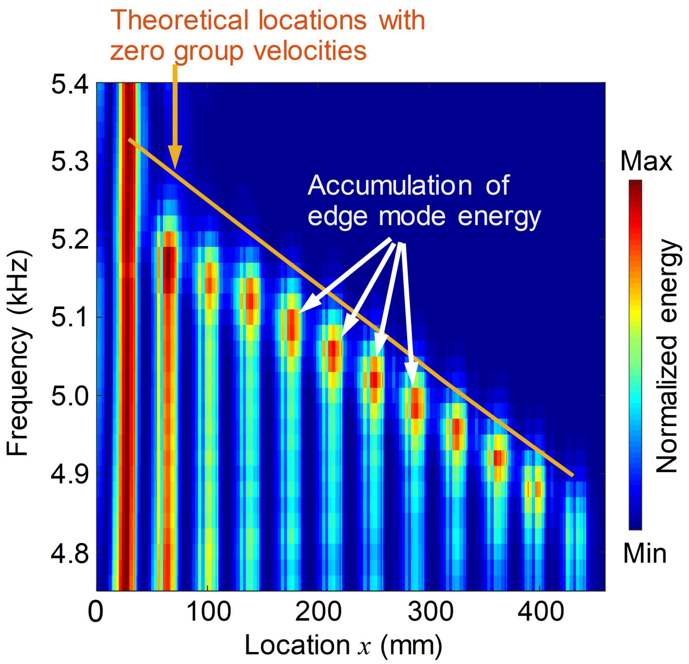

Supplementary Figure 19 | Demonstration of rainbow edge waves in chirped valley phononic crystals. This figure presents the simulation wave energy distribution with respect to the location x along the edge of the chirped valley phononic crystals (PCs) and the frequency f. The energy distribution clearly shows rainbow edge waves. As the wave frequency increases, the wave propagation distance decreases. The locations at which waves stop forward propagation agree well with the prediction based on the theoretical locations with zero group velocity. In addition, high wave energy is accumulated in the low group velocity region. With the decrease of the wave frequency, the wave propagation distance increases and the locations with accumulated energy shift to the right side of the PCs.

Supplementary Notes

Supplementary Note 1 Experimental demonstrations of the frequency-dependent acoustic routing

Frequency-dependent acoustic routing using hybrid channel-cavity PCs is visually demonstrated through acoustic pressure field scanning (Supplementary Figure 6 and Supplementary Figure 7). When all the cavities have the same depth of 11.5 mm and the channel height h_c is 10 mm, at 4.1 kHz near the Dirac degeneracy, the equifrequency contours (EFCs) exhibit multiple triangle-like shapes around the K and K′ points (Supplementary Figure 6b). This feature offers the capability for spatially splitting acoustic waves carrying different pseudospins[3]. For example, when incident acoustic waves, generated by a loudspeaker at the lower side of the phononic crystals (PCs), contain a wide range of momentum components, the forward-propagating states around the K and K′ points will be excited simultaneously, as shown in Supplementary Figure 6. Hence, the incident waves at the two states will travel through different routes along the directions of group velocities $\mathbf{v}_{g1}$ and $\mathbf{v}_{g3}$, which are predicted by the EFCs[4, 5] (Supplementary Figure 6b). As shown in the measured acoustic pressure field in Supplementary Figure 6e, the incident waves generated by a loudspeaker are separated into two beams corresponding to the two forward-propagating states around K and K′ points. This effect is analogous to the valley Hall effect observed in MoS_2 transistors[6], where the sign of Hall voltage depends on the helicity of incident light.

At frequencies away from the Dirac degeneracy, for example 4.7 kHz, the EFC changes to a hexagon-like shape (Supplementary Figure 6c). As the frequency increases, the EFC gradually shrinks. Such unique EFC allows for confining and guiding acoustic waves using the proposed PCs. The experimentally acquired acoustic pressure field (Supplementary Figure 6f) visually

shows that incident waves from a loudspeaker (acting as a point source) are transformed to a highly confined beam. Moreover, the beam direction agrees well with the direction of group velocity $\mathbf{v}_{g2}$ predicted by the EFC.

To evaluate the bandwidth of frequency-dependent wave routing, transmission coefficients at three receivers R1-R3 (illustrated in Supplementary Figure 6a) are compared in Supplementary Figure 6d. At 4.1 kHz, the transmission coefficients for R1 and R3 are much higher than the coefficient for R2 because of the beam splitting effect. On the other hand, the transmission coefficients for R1 and R3 become smaller than the coefficient for R2 at 4.7 kHz, due to the wave confinement and guiding. Besides guiding and splitting waves coming from the lower side of the PCs shown in Supplementary Figure 6, incident acoustic waves from the left side of the PCs can be guided and split as demonstrated by the experimental results in Supplementary Figure 7.

As proven by the experimental results, different frequency components can be split and guided along distinct routes. Moreover, the routing effect can be realized at desired frequencies by carefully changing the height h_c, since the Dirac frequency can be conveniently tuned by changing h_c. The presented PCs support frequency-dependent acoustic routing and can potentially be used for frequency splitters[7, 8], wave logics[9-12], and information processing[13, 14].

Supplementary Note 2 Experimental demonstrations of the modulation of acoustic refraction

The refraction of acoustic (or electromagnetic) waves can be controlled by engineering the equifrequency contours (EFCs) of phononic[15-17] (or photonic[18-20]) crystals. By using PCs to precisely control the propagation of acoustic waves, negative refraction and focusing of acoustic waves has been demonstrated[15, 16]. Here, we experimentally demonstrate the capability of our PCs for modulating acoustic refraction from positive to negative simply by adjusting cavity depths. Such refraction modulation can be predicted through EFC analysis[4] using the conservation of momentum. Supplementary Figure 8a shows the experimental setup for positive refraction between PCs with different cavity depths. The cavity depths on the left and right sides of the flat interface are uniformly distributed (i.e., $h_1 = h_2$) and are 10.5 and 1 mm, respectively. The refraction behavior predicted by the EFC analysis is given in Supplementary Figure 8b. On the left side of the interface, acoustic waves generated by a loudspeaker will be locked at the forward-propagating state near the K point with the group velocity $\mathbf{v}_{gI}$. On the right side of the interface, since the cavity depths are very small, the anisotropy introduced by cavities in honeycomb lattices becomes very weak and the EFC degenerates to a nearly isotropic contour. The EFC analysis (Supplementary Figure 8b) predicts that forward-propagating waves will be refracted positively at the interface with the group velocity of refraction waves being $\mathbf{v}_{gR}$. The acquired experimental pressure field at 4.3 kHz (Supplementary Figure 8c) agrees well with the theoretical analysis, which clearly shows that waves with an incident angle of 30 degrees are refracted positively with a refraction angle of 43 degrees.

By gradually increasing cavity depths of PCs on the right side of the interface, the anisotropy induced by cavities in honeycomb lattices becomes stronger, and thus the EFC

transforms from a quasi-isotropic contour to an anisotropic contour. Such transformation opens the opportunity for modulating acoustic refraction by changing the cavity depths. Supplementary Figure 8d shows the experimental setup for negative refraction. The cavity depth of PCs on the right side of the interface are increased to 13.5 mm, and accordingly the EFC transforms to a hexagon-like shape (Supplementary Figure 8e). The EFC analysis predicts that forward-propagating waves will be refracted negatively at the interface. As predicted, the acquired experimental pressure field (Supplementary Figure 8f) shows that incident waves are refracted negatively with a refraction angle of −30 degrees.

Supplementary Note 3 Generation and frequency tuning of the WAW-type edge mode

To demonstrate that our valley PCs can support edge waves in a waveguide, numerical simulations in COMSOL Multiphysics are performed. Supplementary Figure 9a gives a schematic of the model setup for simulating the edge mode of waveguide acoustic waves (WAWs). The valley PCs are composed of a top channel with a height h_c of 10 mm and an array of cavities with the same diameter d of 8.7 mm at the bottom. In the type A and B PCs, the depths for deep and shallow cavities are 12.5 and 10.5 mm, respectively. The lattice constant a is 36.5 mm. There is a Z-shaped interface between the type A and B PCs. To generate incident acoustic waves, the plane wave radiation condition is applied on the boundary at the left end of the interface. Impedance boundary conditions are applied on the side boundaries of the channel with the impedance $Z = \rho_{air}c_{air}$, where ρ_{air} and c_{air} are the air density and sound speed. The rest boundaries are considered as hard walls.

The simulation pressure fields are given in Supplementary Figure 9e-g, which are for three representative cross sections of the valley PCs, including cross sections at z = 10 mm, z = 0 mm, and y = 295 mm. The simulation pressure fields show that a valley Hall edge mode is generated and propagates along the Z-shaped interface. The pressure field in the plane z = 10 mm at the top of the channel (Supplementary Figure 9e) is nearly the same as that in the plane z = 0 mm at the bottom of the channel (Supplementary Figure 9f). The pressures in bottom cavities couple with the pressures in the horizontal channel (Supplementary Figure 9g). The generated edge waves can be considered as waveguide acoustic waves that are confined in a waveguide composed of a horizontal channel and an array of cavities. In our study, the edge mode propagating in a waveguide is referred as a 'WAW-type edge mode'.

To demonstrate the capability of our valley PCs for tuning the frequency of WAW-type edge waves, simulations are performed at different channel heights from 1 to 30 mm. The simulation pressure fields in Supplementary Figure 10 clearly show that the frequencies of WAW-type edge waves can be tuned from 2.55 kHz at the channel height of 1 mm to 4.66 kHz at the channel height of 30 mm. The generated WAW-type edge waves at different frequencies are confined and guided along a Z-shaped path. Although the pressure fields for edge waves are at different frequencies, their phase patterns are nearly identical. This means edge waves at those different frequencies have nearly the same wavelength and wavenumber. Moreover, the edge mode frequency gradually increases with the increase of the channel height. The simulation results prove that our valley PCs can tune the frequency of WAW-type edge waves without changing the wavenumber by adjusting the channel height. This interesting capability is meaningful for broadband operation of the edge waves.

Supplementary Note 4 Robust routing and frequency tuning of WAW-type edge modes

The topological protection with pseudospins allows robust transport of the valley Hall edge mode without obvious backscattering. Thus, it becomes possible to efficiently route the edge mode along different paths with sharp corners by engineering the interface of valley topological PCs. We experimentally demonstrate two routes as proof-of-concept results, one for transporting acoustic waves along a Z-shaped interface with two sharp corners (Fig. 3a) and the other for 180 degrees turning of acoustic waves (Fig. 3b). For both cases, the channel height is set to 10 mm, so that a WAW-type edge mode can be excited. The sharp corners are introduced at least two wavelengths away from the source in order to ensure that the generated bulk waves are highly suppressed in the PCs and the edge mode at sharp corners can be clearly observed without strong interference with the bulk waves. The measured acoustic pressure fields (Fig. 3e and f) show that the generated edge waves are efficiently guided along interfaces without obvious scattering even at sharp corners. For quantitative characterization, the transmission coefficients for waves transporting along the interface (R1) and propagating along a straight path (R2) are compared in Fig. 3i for the first route and Fig. 3j for the second route. The comparison shows that the transmission at R1 is much higher than that at R2 in the frequency range 4.15 ~ 4.55 kHz for both routes. The maximum contrasts are nearly 18 and 33 dB for the first and second routes, respectively. We also compare the experimental transmission spectra with simulation results (Supplementary Figure 9j). Both the numerical and experimental results for receivers off the interface have transmissions lower than −10 db in the frequency range 4.22~4.60 kHz, while both results show transmissions above −10 db in the frequency range 4.22~4.46 kHz for receivers on the interface. The maximum experimental transmission is near −1 dB at 4.27 kHz,

which is close to the maximum simulation transmission 0 dB at 4.40 kHz. These experimental and numerical results confirm the robustness and efficiency of transporting acoustic waves along complex routes with sharp corners in our system.

Based on the acoustic valley Hall edge mode, we further demonstrate spin-locked wave division with a four-port topological junction. As shown in Fig. 3c, the entire structure is divided into four sections by interfaces with four branches. Using a loudspeaker placed at the left port, an edge mode with a positive group velocity and a negative wavenumber k_{n+} is excited. The excited edge mode is locked by pseudospin + and pseudospin − in the top-left and bottom-left sections of the PCs (Fig. 3g). Because of the pseudospin-dependent propagation, the left and right branches only support inward wave propagation (toward the four-port junction) and the top and bottom branches only support outward wave propagation (away from the four-port junction). As verified by the experimental pressure field (Fig. 3g), at the four-port junction, waves are nearly equally divided into two parts that propagate upward and downward along the top and bottom branches, respectively. The transmission coefficients at R1-R3 (bottom, right, and top ports) are compared in Fig. 3k. It can be seen that the transmission coefficients for R1 and R3 are very close and are both much stronger than the coefficient for R2 in the range 4.15 ~ 4.55 kHz.

A unique feature of our acoustic system is that the edge mode frequency can be tuned while locking the real wavenumber part and in-plane phase pattern. Finite element simulations are performed to demonstrate this tunable effect (Supplementary Note 3). The simulation pressure fields in Supplementary Figure 10 show that for different channel heights in the range 1~30 mm, WAW-type edge waves can be generated at different frequencies and guided along a Z-shaped interface between type A and B PCs. As the channel height increases, the edge mode frequency gradually increases from 2.55 to 4.66 kHz. Moreover, the in-plane phase patterns of edge waves

for all the cases are nearly identical, which means edge waves at different frequencies have nearly the same wavenumber. These findings prove that our valley PCs can tune the edge mode frequency while locking the wavenumber and the in-plane phase pattern. For further verification of the frequency tuning, experiments are performed using the PC configuration with a straight edge (Fig. 3d). The scanned pressure field in Fig. 3h shows that edge waves are generate at 4.3 kHz and guided straightly along the edge of valley PCs when the channel height is 10 mm. We further acquire a 2D representation (Fig. 3l) of normalized pressure amplitudes for transmitted waves through the straight edge versus frequency and channel height. The 2D representation shows that the frequency of transmitted edge waves gradually increases from 2.6 to 4.3 kHz with the increase of channel height from 0.4 to 10.4 mm, which experimentally verifies the frequency tuning capability.

The above experimental and numerical studies confirm the capability of our valley PCs for controlling WAW-type edge modes. We have also experimentally demonstrated robust topological switches for edge modes (Supplementary Figure 11). The experimental results show a maximum transmission contrast between the switch-on and switch-off states of nearly 30 dB and confirm that the topological switch is immune to local defects in the system, as expected.

Supplementary Note 5 Experimental demonstrations of topological switches for acoustic waves

The one-way propagation with pseudospins endows the edge mode with topological protection against backward scattering as long as the pseudospins are preserved. By taking advantage of the topologically protected edge mode, it becomes possible to realize robust topological switches with our PCs. As shown in Supplementary Figure 11, the switching effect for acoustic waves is experimentally demonstrated by turning on/off the valley Hall edge mode. When all the PCs are type B (Supplementary Figure 11a), the edge mode is off. Acoustic waves generated by a loudspeaker at 4.3 kHz are blocked by the PCs (Supplementary Figure 11e), due to the existence of a complete band gap. By changing the cavity depths and introducing type A PCs (Supplementary Figure 11b), a type A-B interface is formed to switch on the edge mode. As a result, an edge mode is excited and acoustic waves at 4.3 kHz transmit through the A-B interface with low loss (Supplementary Figure 11f). The transmission coefficients at switch-on and -off states are further compared in Supplementary Figure 11i. It can be seen that in the frequency range 4.15 ~ 4.55 kHz, the transmission coefficient at the switch-on state is much higher than that at the switch-off state. The maximum transmission contrast is nearly 30 dB, indicating high contrast of the delivered acoustic energy between the on and off states.

To demonstrate the robustness of the topological switch, we deliberately introduce two types of defects to the A-B interface. In the experiments, the defects are introduced at least two wavelengths away from the source, in order to ensure that the bulk waves generated from the wave source are highly suppressed and don't interact with defects. In addition, we want to ensure the edge mode at defects can be clearly observed without strong interference with bulk waves. For the first case, two cavities (marked as red stars in Supplementary Figure 11c) on the A-B

interface are full of water. The measured pressure field in Supplementary Figure 11g is nearly the same as the pristine result in Supplementary Figure 11f. For the second case, a scatterer made of an acrylic block (marked as a red rectangular in Supplementary Figure 11d) is placed in the middle of the A-B interface. The size of the scatterer is 15 mm by 20 mm and its height being 10 mm, which fully occupies the waveguide in the z axis. Such block is able to strongly scatter the incoming acoustic waves in an ordinary waveguide. The measured pressure field in Supplementary Figure 11h visually shows that the excited forward-propagating waves can bypass the acrylic block without undesired scattering, thanks to the robustness of the edge mode. The corresponding transmission coefficients for the pristine and two cases with different defects are compared in Supplementary Figure 11i. It can be seen that trends of transmission coefficients with respect to frequencies for the two defected cases closely follow the trend for the pristine case. Moreover, the maximum coefficients for them are nearly the same. These agreements confirm the robustness of the topological switch that the acoustic wave transport is immune to local defects.

Supplementary Note 6 Generation and frequency tuning of the SAW-type edge mode

To demonstrate that our valley PCs can support the edge mode of surface acoustic waves along a structure's surface, numerical simulations are performed in COMSOL Multiphysics. Supplementary Figure 12a shows a schematic of the model setup for simulating the edge mode of surface acoustic waves (SAWs). The model has an array of cavities with the same diameter d of 8.7 mm. In the type A and B PCs, the depths for deep and shallow cavities are 12.5 and 10.5 mm, respectively. The lattice constant a is 36.5 mm. Between the type A and B PCs, there is a Z-shaped interface. To generate incident acoustic waves, the plane wave radiation condition is applied on a boundary at the left end of the interface. Impedance boundary conditions are applied on the top and side boundaries of the channel with the impedance Z being $\rho_{air}c_{air}$, where ρ_{air} and c_{air} are the air density and sound speed. Since the applied impedance at the top of the channel matches with that of the background air, the effective height of the channel can be considered as infinity. Hence, we can use the model for simulating SAWs.

The simulation pressure fields are given in Supplementary Figure 12e-g, which are for three representative cross sections of the valley PCs, including cross sections at z = 50 mm, z = 0 mm, and y = 295 mm. The simulation pressure fields show that a valley Hall edge mode is generated and propagates along the Z-shaped interface. The pressure amplitude near the bottom cavities (Supplementary Figure 12f) is much larger than the amplitude in areas that are far from the bottom cavities (Supplementary Figure 12e). As the distance to the bottom cavities increases, the pressure amplitude drops rapidly, as shown in Supplementary Figure 12g. These results clearly show that an edge mode propagating along the structure's surface (an array of cavities) is generated. This edge mode is referred as a 'SAW-type edge mode' in this study.

To demonstrate the capability of our valley PCs for tuning the frequency of SAW-type edge waves, simulations are performed for five configurations with different cavity depths. The simulation pressure fields in Supplementary Figure 14 clearly show the generated SAW-type edge waves at different frequencies are confined and guided along a Z-shaped path. Supplementary Figure 14a shows the pressure field for the case that the depths of deep and shallow cavities are 12.5 and 10.5 mm. In other cases, the depth change H_δ is applied on all the deep and shallow cavities and their depths become $h_{dp} + H_\delta$ and $h_{sl} + H_\delta$. Supplementary Figure 14b to Supplementary Figure 14e are simulations results for cases that the depth change H_δ decreases from −0.5 to −2 mm. By comparing the results in Supplementary Figure 14a-e, it can be found that the frequencies of SAW-type edge waves can be tuned from 4.7 kHz to 5.11 kHz by reducing the cavity depths. Although the frequencies of pressure fields for edge waves increase, the phase patterns in all the fields are nearly identical. This means the SAW-type edge waves at those different frequencies have nearly the same wavelength and wavenumber. The simulation results prove that our valley PCs can tuning the frequencies of SAW-type edge waves without changing the wavenumber by simply adjusting the cavity depths.

Supplementary Note 7 Rainbow SAW-type edge waves in chirped valley PCs

Valley PCs support the valley Hall edge mode that is confined in and propagates along the interface between two types of PCs with inversion symmetry breaking. Most studies focus on the demonstrations of edge modes in different acoustic systems. Few studies investigate the potentials of spatially tailoring the dispersion of the edge mode and modulating the edge mode in both the space and frequency domains. Here, we present chirped valley PCs with gradient cavity depths along the interface between two types of PCs. Our study shows that the dispersion curves of edge waves can be spatially modulated. Moreover, edge waves at different frequencies can be spatially separated with the energy accumulated at different locations along the interface, manifested as the "acoustic rainbow" of edge waves.

In this Supplementary Note, edge waves in chirped valley PCs are investigated from two aspects, their dispersion curves and the propagation of edge waves. Supplementary Figure 15a shows a 3D schematic of the chirped valley PCs, which contain A-B type supercells with a straight interface (blue line). Supplementary Figure 15b is a section view of the valley PCs from the plane A-A to the $+y$ direction, which illustrates that the cavity depths in supercells gradually increase along the $+x$ direction. Supplementary Figure 15c is a top view of the chirped valley PCs. The blue and grey circles illustrate locations of shallow and deep cavities. The depths of shallow and deep cavities in the mth column are $h_{\text{sl},m}$ and $h_{\text{dp},m}$, which linearly increase with the increase of column index m, following the relations $h_{\text{sl},m} = h_{\text{sl},1} + (m-1)h_\delta$ and $h_{\text{dp},m} = h_{\text{dp},1} + (m-1)h_\delta$. The depths of the shallow and deep cavities in the first column are $h_{\text{sl},1} = 8.58$ mm and $h_{\text{dp},1} = 10.58$ mm, respectively. The step size h_δ is 0.08 mm. The lattice constant a and cavity diameter d are 36.5 and 8.7 mm, respectively. The plane wave radiation condition is applied on a boundary at the left end of the interface. Impedance boundary conditions are applied on the top

and side boundaries of the channel with the impedance Z being $\rho_{air}c_{air}$, where ρ_{air} and c_{air} are the air density and sound speed. Since the applied impedance matches with that of the background air, the effective height of the channel can be considered as infinity. The rest boundaries of the valley PCs are set as hard walls. With these boundary conditions, the SAW-type edge waves can be investigated.

The derived dispersion curves for the chirped valley PCs are given in Supplementary Figure 16 showing the variations of edge mode dispersion curves along the straight interface in the chirped valley PCs. Supplementary Figure 16b-d are wavenumber-frequency dispersion relations for supercells in the 1st, 13th, and 25th columns with total depth changes H_δ of 0, 0.96, and 1.92 mm. These dispersion relations show that an edge mode (blue dotted line) presents. Supplementary Figure 16e compares multiple wavenumber-frequency dispersion curves for different cavity depth changes H_δ from −0.08 to 1.92 mm. It can be seen that the edge mode frequency gradually decreases with the increase of depth change H_δ. Supplementary Figure 16f further compares the group velocity c_g dispersion curves for different cavity depth changes H_δ from −0.08 to 1.92 mm. These group velocity curves show that the edge mode has a cutoff frequency f_{cut}. At the cutoff frequency, the group velocity is zero and above this frequency edge waves disappear. With the decrease of the depth change H_δ, the maximum group velocity becomes larger and the cutoff frequency f_{cut} increases. Moreover, the group velocities are much smaller than the wave speed 343 m/s in air, which means the edge mode can be used to effectively delay acoustic waves.

Supplementary Figure 17a plots variations of group velocities with respect to the location x long the edge of the chirped valley PCs at four selected frequencies of 4.92, 5.00, 5.06, and 5.12 kHz. It can be seen that group velocities gradually slow down along the $+x$ direction and finally

reach zero at the locations marked with colored arrows. As the frequency decreases, the x location with the zero group velocity increases, which means waves can propagate further along the $+x$ direction at a lower frequency.

To investigate the propagation of edge waves in the chirped valley PCs, simulations are performed in COMSOL Multiphysics with the model given in Supplementary Figure 15a. The simulation energy fields of SAW-type edge waves in chirped valley PCs at six selected frequencies of 4.84, 4.92, 4.98, 5.06, 5.12, and 5.20 kHz are given in Supplementary Figure 18a-f, respectively. The energy distributions show that SAW-type edge waves are generated at different frequencies and confined in the edge of the chirped valley PCs. Waves propagate further at lower frequencies. Moreover, at different frequencies, we can observe accumulations of wave energy at different locations, which correspond to the low group velocity regions. The wave energy distributions along a straight line at the center of the edge in the chirped valley PCs are extracted from the simulation fields and plotted in Supplementary Figure 17b. By comparing the energy distributions and the group velocities in Supplementary Figure 17a, it can be found that the locations at which the wave energy drops to zero agree well with the locations with zero group velocity marked with colored arrows. Therefore, we can use the group velocity curve in Supplementary Figure 17a to predict the locations, at which waves stop forward propagation.

To further analyze the frequency-dependent energy localization, numerical simulations are performed in a frequency band from 4.76 to 5.4 kHz with a step of 0.02 kHz. The acoustic energy of edge waves along a line at the center of the edge is extracted. Supplementary Figure 19 plots the extracted energy of edge waves as a 2D representation, which shows the energy distribution with respect to the location x along the interface of the chirped valley PCs and the simulation frequency. The energy distribution in Supplementary Figure 19 shows that the wave

propagation distance decreases as the wave frequency increases. The locations at which waves stop propagating forward agree well with the prediction based on the zero group velocity. In addition, accumulation of edge mode energy presents in the low group velocity region. As the frequency increases, the location with accumulated energy shifts to the left side of the interface, manifested as the rainbow edge waves.

Supplementary References

1. Lu JY, Qiu CY, Ye LP, Fan XY, Ke MZ, Zhang F, Liu ZY. Observation of topological valley transport of sound in sonic crystals. *Nat Phys* **13**, 369-374 (2017).
2. Zhu H, Liu TW, Semperlotti F. Design and experimental observation of valley-Hall edge states in diatomic-graphene-like elastic waveguides. *Phys. Rev. B* **97**, 174301 (2018).
3. Ye LP, Qiu CY, Lu JY, Wen XH, Shen YY, Ke MZ, Zhang F, Liu ZY. Observation of acoustic valley vortex states and valley-chirality locked beam splitting. *Phys. Rev. B* **95**, 174106 (2017).
4. He H, Qiu C, Ye L, Cai X, Fan X, Ke M, Zhang F, Liu Z. Topological negative refraction of surface acoustic waves in a weyl phononic crystal. *Nature* **560**, 61 (2018).
5. Gao F, Xue HR, Yang ZJ, Lai KF, Yu Y, Lin X, Chong YD, Shvets G, Zhang BL. Topologically protected refraction of robust kink states in valley photonic crystals. *Nat Phys* **14**, 140-144 (2018).
6. Radisavljevic B, Radenovic A, Brivio J, Giacometti V, Kis A. Single-layer MoS_2 transistors. *Nat Nanotechnol* **6**, 147-150 (2011).
7. Semperlotti F, Zhu H. Achieving selective interrogation and sub-wavelength resolution in thin plates with embedded metamaterial acoustic lenses. *J. Appl. Phys.* **116**, 054906 (2014).
8. Bozhko A, Sanchez-Dehesa J, Cervera F, Krokhin A. Redirection and splitting of sound waves by a periodic chain of thin perforated cylindrical shells. *Phys. Rev. Appl.* **7**, 064034 (2017).
9. Bringuier S, Swinteck N, Vasseur JO, Robillard JF, Runge K, Muralidharan K, Deymier PA. Phase-controlling phononic crystals: Realization of acoustic Boolean logic gates. *J. Acoust. Soc. Am.* **130**, 1919-1925 (2011).
10. Li F, Anzel P, Yang J, Kevrekidis PG, Daraio C. Granular acoustic switches and logic elements. *Nat. Commun.* **5**, 6311 (2014).
11. Zhang T, Cheng Y, Guo JZ, Xu JY, Liu XJ. Acoustic logic gates and boolean operation based on self-collimating acoustic beams. *Appl. Phys. Lett.* **106**, 113503 (2015).
12. Zhu Y, Gu ZM, Liang B, Yang J, Yang J, Yin LL, Cheng JC. Asymmetric sound transmission in a passive non-blocking structure with multiple ports. *Appl. Phys. Lett.* **109**,

103504 (2016).

13. Xia JP, Jia D, Sun HX, Yuan SQ, Ge Y, Si QR, Liu XJ. Programmable coding acoustic topological insulator. *Adv. Mater.* **2018**, 1805002 (2018).
14. Yu SY, Sun XC, Ni X, Wang Q, Yan XJ, He C, Liu XP, Feng L, Lu MH, Chen YF. Surface phononic graphene. *Nat. Mater.* **15**, 1243-1247 (2016).
15. Yang SX, Page JH, Liu ZY, Cowan ML, Chan CT, Sheng P. Focusing of sound in a 3D phononic crystal. *Phys. Rev. Lett.* **93**, 024301 (2004).
16. Sukhovich A, Jing L, Page JH. Negative refraction and focusing of ultrasound in two-dimensional phononic crystals. *Phys. Rev. B* **77**, 014301 (2008).
17. Zhang XD, Liu ZY. Negative refraction of acoustic waves in two-dimensional phononic crystals. *Appl. Phys. Lett.* **85**, 341-343 (2004).
18. Berrier A, Mulot M, Swillo M, Qiu M, Thylen L, Talneau A, Anand S. Negative refraction at infrared wavelengths in a two-dimensional photonic crystal. *Phys. Rev. Lett.* **93**, 073902 (2004).
19. Takeda H, Yoshino K. Tunable refraction effects in two-dimensional photonic crystals utilizing liquid crystals. *Phys. Rev. E* **67**, 056607 (2003).
20. Cubukcu E, Aydin K, Ozbay E, Foteinopoulou S, Soukoulis CM. Negative refraction by photonic crystals. *Nature* **423**, 604-605 (2003).
